# Differences in gastrointestinal hormones and appetite ratings between individuals with and without obesity—A systematic review and meta‐analysis

**DOI:** 10.1111/obr.13531

**Published:** 2022-11-23

**Authors:** Marthe Isaksen Aukan, Silvia Coutinho, Sindre Andre Pedersen, Melanie Rae Simpson, Catia Martins

**Affiliations:** ^1^ Obesity Research Group, Department of Clinical and Molecular Medicine, Faculty of Medicine Norwegian University of Science and Technology (NTNU) Trondheim Norway; ^2^ Centre of Obesity and Innovation (ObeCe), Clinic of Surgery St. Olav University Hospital Trondheim Norway; ^3^ Department of Public Health Nutrition at the Institute of Basic Medical Sciences, Faculty of Medicine University of Oslo (UiO) Oslo Norway; ^4^ Library Section for Research Support, Data and Analysis Norwegian University of Science and Technology (NTNU) Trondheim Norway; ^5^ Department of Public Health and Nursing Norwegian University of Science and Technology Trondheim Norway; ^6^ Clinical Research Unit Central Norway St. Olavs Hospital Trondheim Norway; ^7^ Department of Nutrition Sciences the University of Alabama at Birmingham (UAB) Birmingham Alabama USA

**Keywords:** appetite, ghrelin, obesity, PYY

## Abstract

Determining if gastrointestinal (GI) hormone response to food intake differs between individuals with, and without, obesity may improve our understanding of obesity pathophysiology. A systematic review and meta‐analysis of studies assessing the concentrations of GI hormones, as well as appetite ratings, following a test meal, in individuals with and without obesity was undertaken. Systematic searches were conducted in the databases MEDLINE, Embase, Cochrane Library, PsycINFO, Web of Science, and ClinicalTrials.gov. A total of 7514 unique articles were retrieved, 115 included in the systematic review, and 70 in the meta‐analysis. The meta‐analysis compared estimated standardized mean difference in GI hormones' concentration, as well as appetite ratings, between individuals with and without obesity. Basal and postprandial total ghrelin concentrations were lower in individuals with obesity compared with controls, and this was reflected by lower postprandial hunger ratings in the former. Individuals with obesity had a lower postprandial concentration of total peptide YY compared with controls, but no significant differences were found for glucagon‐like peptide 1, cholecystokinin, or other appetite ratings. A large methodological and statistical heterogeneity among studies was found. More comprehensive studies are needed to understand if the differences observed are a cause or a consequence of obesity.

**Abbreviations:**
AUC: area under the curve
BMI: body mass index
CCK: cholecystokinin
DTE: desire to eat
GI: gastrointestinal
GLP‐1: glucagon‐like peptide 1
PFC: prospective food consumption
PYY: peptide YY

## 1 INTRODUCTION

Obesity is a chronic relapsing disease,^1^ characterized by excessive fat accumulation and defined as a body mass index (BMI) ≥ 30 kg/m^2^ (with cutoffs varying among ethnicities).^2^ The prevalence of obesity has increased dramatically over the past five decades resulting in socioeconomic challenges and public health issues. Obesity is associated with reduced quality of life and poorer mental health, increased risk of noncommunicable diseases, and shortened life expectancy.^3^ Thus, obesity represents one of the greatest global health problems of our times.

The causes of obesity are multifactorial, and genetics play an important role.^4^ The contribution of the gut–brain axis and the underpinnings of homeostatic body weight regulation have been frequently investigated in the context of obesity pathophysiology.^5^ Ingestion of food leads to the stimulation, or inhibition, of the secretion of different hormones from distinctive sites of the gastrointestinal (GI) tract.^6^ These peripheral signals have the potential to modulate food intake and long‐term body weight homeostasis, both via hormonal and vagal pathways.^7^ Ghrelin is the only known peripheral hormone with orexigenic properties.^8^ Its plasma concentration peaks during fasting and in anticipation of the upcoming meal and declines in the postprandial period.^9^ On the other hand, as macronutrients interact with receptors of enteroendocrine cells, several anorexic peptides, including glucagon‐like peptide‐1 (GLP‐1), peptide YY (PYY), and cholecystokinin (CCK), are secreted from the GI tract, promoting satiation and satiety.^10^, ^11^, ^12^

Obesity has been shown to be associated with alterations in the secretion of several GI hormones, increased fasting gastric volume, accelerated gastric emptying, and decreased satiety.^13^, ^14^, ^15^ Many studies have measured the basal and postprandial plasma concentrations of GI hormones in individuals with obesity, and the majority reports lower basal and postprandial plasma concentrations of ghrelin compared with individuals without obesity.^15^, ^16^, ^17^ However, results regarding satiety peptides are inconclusive, most likely due to different hormonal fractions being measured.^14^, ^16^, ^18^ Understanding if obesity is, or not, associated with alterations in the secretion of GI hormones, and subjective appetite feelings, will improve our knowledge on the pathophysiology of this chronic disease. Therefore, this systematic review and meta‐analysis aimed to compare basal and postprandial plasma concentrations of GI hormones, as well as subjective appetite ratings, between individuals with and without obesity.

## 2 METHODS

This systematic review and meta‐analysis is registered in PROSPERO (PROSPERO 2020 CRD42020161552) (https://www.crd.york.ac.uk/prospero/display_record.php?ID=CRD42020161552). The PRISMA statement for systematic reviews and meta‐analyses was followed.^19^

### 2.1 Literature search

A structured database search was conducted in MEDLINE, Embase, Cochrane Library, PsycINFO, Web of Science, and ClinicalTrials.gov. The query involved a combination of thesaurus‐ and free‐text terms optimized to capture studies comparing appetite/appetite markers between individuals with and without obesity. The search strategies excluded studies focusing exclusively on nonadults, or animals, and publication types like comments, editorials, or news. The searches were also restricted to studies written in English, Norwegian, Swedish, Danish, Portuguese, French, or Spanish (see Supporting Information for a detailed description of the search strategies adopted in the different databases).

### 2.2 Study selection

Two reviewers (MIA, SC) independently screened titles and abstracts of the identified articles based on the predefined inclusion and exclusion criteria. Results from each reviewer were compared to ensure that exclusions were made on the same basis before screening full text articles. Any disagreements between the reviewers were discussed, and a third reviewer (CM) involved if needed. Full text articles were screened, and assessment of risk of bias was performed for all the included articles in the meta‐analysis.

### 2.3 Eligibility criteria

The database search was conducted based on the following inclusion criteria: A study population of adults with obesity (BMI ≥ 30 kg/m^2^) and a control group without obesity (BMI 18.5–29.9 kg/m^2^); assessing one or several of the following variables in the fasting state and/or after a test meal (using total area under the curve (AUC) as a measure of postprandial response): Plasma or serum concentrations of total or active ghrelin, total or active GLP‐1, total or active PYY, or CCK, and/or appetite ratings of hunger, fullness, desire to eat (DTE), or prospective food consumption (PFC) measured with a visual analog scale.^20^ Postprandial data were not included if appetite measures were taken under infusion of pharmacological agents or hormonal infusion. Exclusion criteria included diabetes or other endocrine disorders known to affect appetite, previous bariatric surgery, and current or recent use of medications known to affect appetite or body weight. Because ketosis is known to affect appetite,^21^ studies were excluded if the appetite measurements were taken while participants were ketotic (defined as a plasma ßeta‐hydroxybutyrate concentration >0.3 mmol/L).

### 2.4 Data extraction

The two reviewers (MIA, SC) extracted the data of all included articles. General characteristics of the participants (i.e., age, sex, BMI, and body composition) were extracted from each article along with energy and macronutrient composition of the test meal used, duration of postprandial period, and frequency of blood sampling/appetite ratings assessment.

### 2.5 Risk of bias assessments

Depending on the study designs, the risk of bias in the articles included in the meta‐analysis was assessed using the Cochrane tools ROB‐2^22^ and ROBINS‐1^23^ for randomized and nonrandomized studies, respectively (Supporting Information). The tools identified to what extent studies addressed the possibility of bias in their design, conduct, and analysis. Any disagreements that arose between the reviewers were resolved through discussion and assistance of a third reviewer when required.

### 2.6 Statistical analysis

The mean and standard deviation for hormone concentrations and appetite ratings in the fasting, and postprandial state (AUC) were extracted. Articles with extreme values (more than 10‐fold larger than the average) were excluded from the meta‐analysis. Articles reporting incremental AUC or calculating total AUC using “0” as basal value were also excluded. When not reported, the standard deviation was calculated from the provided standard error or confidence intervals. Data reported as medians and interquartile range were converted to means and standard deviations (SDs).^24^ If hormonal concentrations were reported in metric units, data were converted to SI units as follows: ghrelin pg/ml × 0.3 = pmol/L, GLP‐1 pg/ml × 0.33 = pmol/L, PYY pg/ml × 0.25 = pmol/L. All values for subjective appetite ratings were converted to millimeters. AUC data were converted to minutes whenever necessary. Some studies reported data on subgroups within the obesity and controls groups, for example, for men and women separately. Prior to inclusion in the meta‐analysis, outcomes for these subgroups were pooled to obtain a single pooled mean and standard deviation within the obesity and control groups separately. For studies including more than one test meal, the meal closest to the balanced dietary recommendations in terms of macronutrient composition was selected. For studies including more than one basal measure, before different infusions of nutrients, a basal value was chosen at random. When two basal values were given before infusion of saline or a hormone, the basal value measured before hormone infusion was selected. The corresponding authors of the respective articles were contacted for further information or clarification when needed. If the missing data were not obtained, the respective article was included in the systematic review, but not in the meta‐analysis.

Meta‐analyses were conducted to compare the obesity and control groups for each outcome, when there were at least three studies. Pooled estimates of standardized mean differences (SMDs) were obtained using a random‐effects model. Statistical heterogeneity was investigated using the *I* ^2^ statistic and a threshold of 75% was considered to represent high heterogeneity.^25^ Evidence of publication bias was assessed by visual inspection of Funnel plots and Egger's test. Analyses were performed using Stata version 16.1 (Stata Corp., College Station, Texas, USA).

## 3 RESULTS

### 3.1 Search result

A total of 13,273 records were obtained by collecting the results from the different databases into a common library. After removing duplicates, 7514 unique records remained. Manual screening of the records, based on title and abstract, identified 163 potentially relevant records. Further full‐text screening of these records identified 115 studies relevant for inclusion. A flow chart of the search results and selection process can be seen in the Supporting Information.

### 3.2 Systematic review

A systematic review comparing concentrations of GI hormones, as well as appetite ratings, between individuals with and without obesity was conducted. A total of 115 articles were included, resulting in the comparison of 22 variables.

#### 3.2.1 Concentrations of GI hormones in the fasting state

##### Basal active ghrelin

Twenty‐four articles compared basal active ghrelin between individuals with obesity and controls. Fifteen studies reported higher basal active ghrelin concentrations in controls,^16^, ^26^, ^27^, ^28^, ^29^, ^30^, ^31^, ^32^, ^33^, ^34^, ^35^, ^36^, ^37^, ^38^, ^39^ whereas seven articles found no differences between groups,^40^, ^41^, ^42^, ^43^, ^44^, ^45^, ^46^ and two reported higher concentrations in individuals with obesity.^43^, ^47^

##### Basal total ghrelin

Fifty‐one articles compared basal total ghrelin between individuals with obesity and controls. Forty articles reported higher basal total ghrelin concentrations in the control group,^14^, ^15^, ^26^, ^31^, ^34^, ^45^, ^48^, ^49^, ^50^, ^51^, ^52^, ^53^, ^54^, ^55^, ^56^, ^57^, ^58^, ^59^, ^60^, ^61^, ^62^, ^63^, ^64^, ^65^, ^66^, ^67^, ^68^, ^69^, ^70^, ^71^, ^72^, ^73^, ^74^, ^75^, ^76^, ^77^, ^78^, ^79^, ^80^, ^81^ whereas 11 articles found no differences between groups.^82^, ^83^, ^84^, ^85^, ^86^, ^87^, ^88^, ^89^, ^90^, ^91^, ^92^

##### Basal active GLP‐1

Sixteen articles compared basal concentrations of active GLP‐1 between individuals with obesity and controls. One study found individuals with obesity to have lower basal concentrations,^93^ 14 studies found no significant differences between groups,^40^, ^43^, ^55^, ^66^, ^94^, ^95^, ^96^, ^97^, ^98^, ^99^, ^100^, ^101^, ^102^, ^103^ and one study found higher basal active GLP‐1 concentrations in individuals with obesity.^52^

##### Basal total GLP‐1

Eighteen studies compared basal total GLP‐1 concentration between individuals with obesity and controls. One study reported lower basal concentrations in individuals with obesity,^104^ 15 studies found no significant differences between groups,^35^, ^42^, ^58^, ^89^, ^102^, ^105^, ^106^, ^107^, ^108^, ^109^, ^110^, ^111^, ^112^, ^113^, ^114^ and two studies reported higher basal total GLP‐1 in individuals with obesity.^103^, ^115^

##### Basal active PYY

Fourteen studies compared basal concentrations of active PYY between individuals with obesity and controls. Four studies reported lower basal active PYY concentrations in individuals with obesity,^45^, ^49^, ^71^, ^116^ and 10 studies found no significant differences between groups.^67^, ^78^, ^83^, ^89^, ^100^, ^104^, ^107^, ^117^, ^118^, ^119^

##### Basal total PYY

Ten studies compared basal concentrations of total PYY between individuals with obesity and controls. Three studies reported lower basal total PYY concentrations in individuals with obesity,^120^, ^121^, ^122^ and seven studies found no significant differences between groups.^42^, ^84^, ^90^, ^117^, ^120^, ^123^, ^124^

##### Basal CCK

Twelve articles assessed basal CCK concentrations. One study reported individuals with obesity to have lower basal CCK concentrations compared with controls,^125^ nine articles found no differences between groups,^52^, ^66^, ^104^, ^107^, ^118^, ^119^, ^126^, ^127^, ^128^ and two studies reported greater basal CCK concentrations in individuals with obesity.^18^, ^101^

#### 3.2.2 Concentrations of GI hormones in the postprandial state

##### Active ghrelin (AUC)

Ten articles assessed postprandial active ghrelin. Five articles reported lower concentrations in individuals with obesity,^16^, ^26^, ^27^, ^35^, ^38^ three articles found no differences between groups,^40^, ^42^, ^116^ and two articles reported individuals with obesity to have higher concentrations.^47^, ^129^

##### Total ghrelin (AUC)

Sixteen articles assessed postprandial total ghrelin. Thirteen articles reported lower concentrations in individuals with obesity^15^, ^17^, ^26^, ^53^, ^54^, ^57^, ^59^, ^63^, ^67^, ^71^, ^78^, ^100^, ^129^ and three articles found no differences between groups.^82^, ^88^, ^130^

##### Active GLP‐1 (AUC)

Nine studies assessed postprandial concentrations of active GLP‐1. Four studies reported lower postprandial concentrations in individuals with obesity^15^, ^16^, ^94^, ^101^ and five studies found no significant differences between groups.^40^, ^97^, ^99^, ^100^, ^131^

##### Total GLP‐1(AUC)

Eight studies measured postprandial concentrations of total GLP‐1. Four studies reported individuals with obesity to have lower postprandial concentrations of total GLP‐1,^102^, ^105^, ^114^, ^122^ and four studies found no significant differences between groups.^42^, ^103^, ^113^, ^115^

##### Active PYY (AUC)

Five articles assessed postprandial concentrations of active PYY. Two studies found that individuals with obesity had lower postprandial concentrations of active PYY compared with controls,^71^, ^116^ whereas three studies found no differences between groups.^67^, ^78^, ^100^

##### Total PYY (AUC)

Seven studies measured postprandial concentrations of total PYY. Four studies reported postprandial total PYY concentrations to be lower in individuals with obesity compared with controls,^14^, ^15^, ^16^, ^121^ whereas three found no differences between groups.^42^, ^130^, ^131^

##### CCK (AUC)

Five studies assessed postprandial concentrations of CCK. One article reported individuals with obesity to have lower postprandial CCK concentrations compared with controls,^16^ three studies found no differences between groups,^101^, ^128^, ^130^ whereas one study reported individuals with obesity to have greater postprandial concentration of CCK.^18^

#### 3.2.3 Appetite ratings in the fasting state

##### Hunger

Eighteen articles assessed hunger ratings in the fasted state. Three studies showed that individuals with obesity reported lower hunger ratings compared with controls,^114^, ^116^, ^132^ whereas 15 studies found no differences between groups.^16^, ^18^, ^31^, ^42^, ^47^, ^58^, ^88^, ^90^, ^103^, ^104^, ^107^, ^113^, ^133^, ^134^, ^135^

##### Fullness

Eighteen articles reported ratings of fullness in the fasted state. Fourteen found no differences between groups,^18^, ^29^, ^42^, ^58^, ^88^, ^90^, ^94^, ^103^, ^104^, ^107^, ^113^, ^133^, ^134^, ^135^ whereas four studies reported greater fullness in individuals with obesity compared with controls.^107^, ^114^, ^116^, ^132^

##### DTE

Nine articles measured DTE in the fasted state. One study reported that individuals with obesity had lower DTE compared with controls^132^ and eight studies found no differences between groups.^16^, ^18^, ^47^, ^88^, ^94^, ^133^, ^134^, ^135^

##### PFC

Nine articles assessed PFC in the fasted state. One study reported individuals with obesity to have a lower PFC compared with controls,^132^ whereas eight studies found no differences between groups.^16^, ^42^, ^58^, ^88^, ^103^, ^113^, ^133^, ^135^

#### 3.2.4 Appetite ratings in the postprandial state

##### Hunger (AUC)

Postprandial hunger ratings were assessed in 16 articles. Two studies reported individuals with obesity to have lower postprandial ratings of hunger compared with controls,^18^, ^88^ whereas 14 studies found no differences between groups.^16^, ^42^, ^47^, ^66^, ^90^, ^103^, ^113^, ^114^, ^116^, ^121^, ^130^, ^133^, ^134^, ^135^

##### Fullness (AUC)

Postprandial fullness ratings were assessed in 16 studies. One article showed that individuals with obesity had lower postprandial ratings of fullness,^114^ whereas 15 studies found no differences between groups.^18^, ^42^, ^66^, ^88^, ^90^, ^94^, ^103^, ^113^, ^114^, ^116^, ^121^, ^130^, ^133^, ^134^, ^135^

##### DTE and PFC (AUC)

Eight studies measured postprandial DTE,^16^, ^18^, ^47^, ^88^, ^94^, ^133^, ^134^, ^135^ and seven studies measured postprandial PFC.^16^, ^42^, ^88^, ^103^, ^113^, ^133^, ^135^ All studies reported no differences in DTE or PFC between individuals with obesity and controls.

### 3.3 Meta‐analysis

**FIGURE 1:**
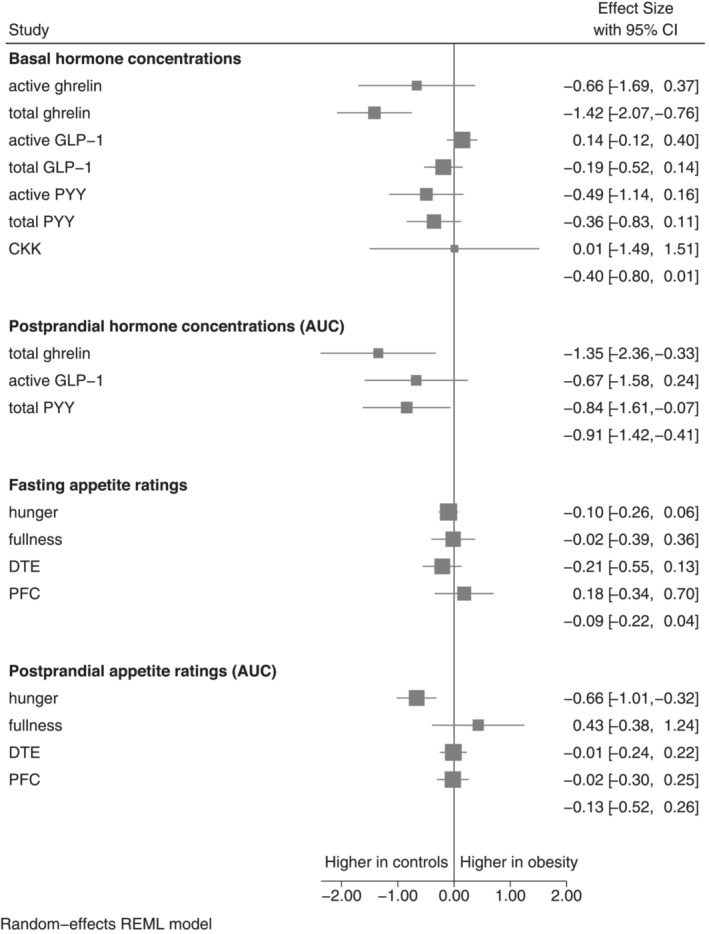
Pooled results from each meta‐analysis for each outcome

A total of 70 articles were included in the meta‐analysis, resulting in the comparison of 18 variables. An overview of the meta‐analysis results can be seen in Table 1, and the pooled results in Figure 1. Twenty‐eight studies were randomized control trials, whereas the remaining had a cross sectional design with a control group. The average BMI of the obesity groups ranged from 29.1 to 57.6 kg/m^2^, whereas control groups had an average BMI range of between 18.5 and 27.6 kg/m^2^. The average age of the obesity groups ranged from 20.8 to 68.5 years, and controls from 20.1 to 68.5 years. The smallest study comprised five individuals with obesity and seven controls,^88^ whereas the largest study comprised 779 individuals with obesity and 1315 controls.^123^ Fourteen studies included only females,^31^, ^41^, ^43^, ^46^, ^55^, ^57^, ^62^, ^63^, ^64^, ^70^, ^90^, ^117^, ^125^, ^136^ 12 studies included only males,^30^, ^47^, ^52^, ^58^, ^84^, ^91^, ^103^, ^104^, ^105^, ^107^, ^113^, ^124^ three studies did not report on sex distribution,^15^, ^69^, ^127^ whereas the remaining studies included a combination of both sexes. When reported, test meals had a varied macronutrient composition and its energy content ranged between 260–632 kcal. Only one study used a single macronutrient loading (75 g glucose).^67^ The AUC period after the test meal varied between 60 and 330 min. For active ghrelin, total GLP‐1, active PYY, and CCK, there were not enough studies (two or less) reporting total AUC concentrations to run the meta‐analysis. Study characteristics and a summary of contributing articles can be seen in the Supporting Information.

**TABLE 1:** Overall meta‐analysis results

Outcome	No. of studies	Reference nr.	SMD (95% CI)	*p* value	I^2^	Egger's test (*p* value)
**Basal hormone concentrations**						
Active ghrelin	16	^14^, ^24^, ^25^, ^27^, ^28^, ^29^, ^30^, ^31^, ^32^, ^38^, ^39^, ^40^, ^41^, ^43^, ^44^, ^45^	−0.66 (−1.69 to 0.37)	0.21	97.98	0.33
Total ghrelin	33	^12^, ^15^, ^24^, ^26^, ^30^, ^41^, ^43^, ^47^, ^49^, ^50^, ^51^, ^52^, ^53^, ^55^, ^56^, ^57^, ^58^, ^60^, ^61^, ^62^, ^63^, ^65^, ^66^, ^67^, ^68^, ^82^, ^83^, ^84^, ^85^, ^86^, ^87^, ^88^, ^89^	−1.42 (−2.07 to −0.76)	<0.001	96.96	0.03
Active GLP‐1	7	^14^, ^38^, ^50^, ^92^, ^93^, ^96^, ^101^	0.14 (−0.12 to 0.40)	0.29	32.81	0.53
Total GLP‐1	13	^40^, ^53^, ^56^, ^87^, ^94^, ^101^, ^102^, ^103^, ^104^, ^106^, ^107^, ^108^, ^111^	−0.19 (−0.52 to 0.14)	0.26	62.35	0.06
Active PYY	8	^43^, ^47^, ^65^, ^87^, ^88^, ^102^, ^105^, ^115^	−0.49 (−1.14 to 0.16)	0.14	85.12	0.35
Total PYY	7	^14^, ^40^, ^82^, ^115^, ^118^, ^121^, ^122^	−0.36 (−0.83 to 0.11)	0.14	90.09	0.60
CKK	7	^14^, ^16^, ^50^, ^102^, ^123^, ^125^, ^134^	0.01 (−1.49 to 1.51)	0.99	96.29	0.16
**Postprandial (AUC) hormone concentrations**						
Total ghrelin	5	^13^, ^15^, ^51^, ^65^, ^86^	−1.35 (−2.36 to −0.33)	0.01	86.16	0.06
Active GLP‐1	4	^13^, ^14^, ^92^, ^95^	−0.67 (−1.58 to 0.24)	0.15	88.92	0.48
Total PYY	3	^13^, ^14^, ^118^	−0.84 (−1.61 to −0.07)	0.03	80.08	0.88
**Fasting appetite ratings**						
Hunger	11	^14^, ^27^, ^29^, ^45^, ^56^, ^88^, ^101^, ^102^, ^105^, ^130^, ^133^	−0.10 (−0.26 to 0.06)	0.23	0.00	0.40
Fullness	9	^14^, ^27^, ^56^, ^88^, ^101^, ^102^, ^105^, ^130^, ^133^	−0.02 (−0.39 to 0.36)	0.94	71.80	0.36
DTE	4	^14^, ^45^, ^130^, ^133^	−0.21 (−0.55 to 0.13)	0.22	54.70	0.95
PFC	5	^14^, ^56^, ^101^, ^130^, ^133^	0.18 (−0.34 to 0.70)	0.49	81.42	0.32
**Postprandial (AUC) appetite ratings**						
Hunger	5	^14^, ^45^, ^86^, ^101^, ^133^	−0.66 (−1.01 to −0.32)	<0.001	41.82	0.08
Fullness	4	^14^, ^86^, ^101^, ^133^	0.43 (−0.38 to 1.24)	0.30	87.63	0.38
DTE	4	^14^, ^86^, ^101^, ^133^	−0.01 (−0.24 to 0.22)	0.91	0.00	0.48
PFC	4	^14^, ^86^, ^101^, ^133^	−0.02 (−0.30 to 0.25)	0.88	13.94	0.46

Abbreviations: AUC, area under the curve; CCK, cholecystokinin: DTE, desire to eat; GLP‐1, glucagon‐like peptide 1; PFC, prospective food consumption; PYY, peptide YY; SMD, standardized mean difference.

#### 3.3.1 Concentrations of GI hormones in the fasting and postprandial states

**FIGURE 2:**
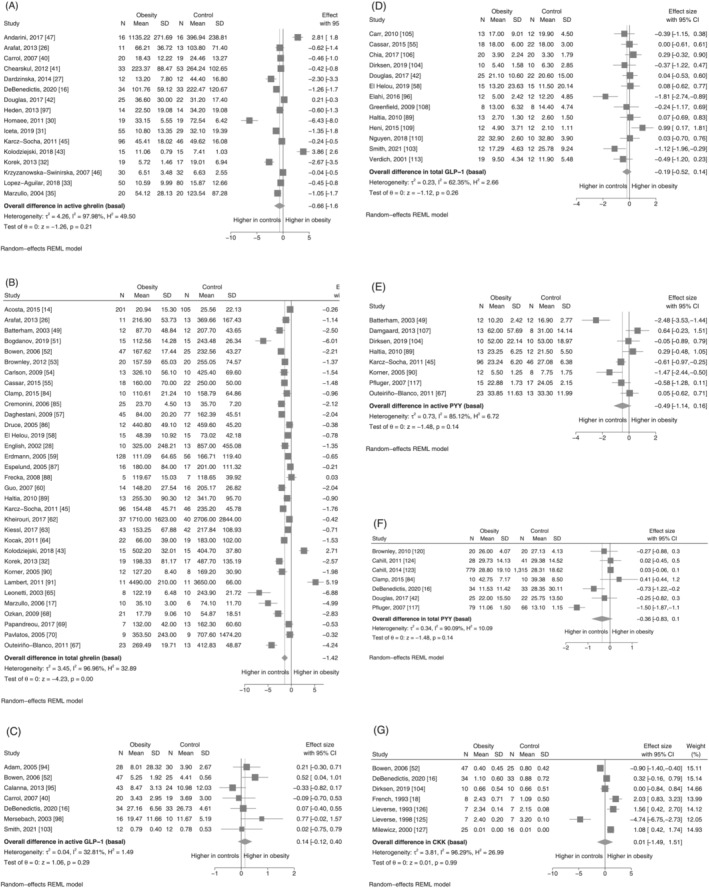
(A–G) Meta‐analysis results for basal concentrations of gastrointestinal hormones

**FIGURE 3:**
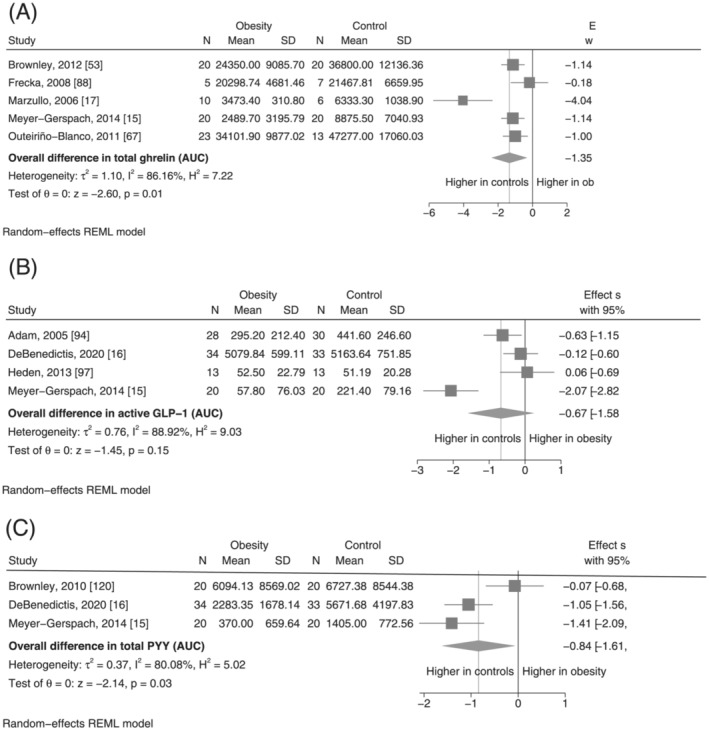
(A–C) Meta‐analysis results for postprandial concentrations of gastrointestinal hormones

Overall, we observed a high degree of statistical heterogeneity between studies investigating the concentration of GI hormones in the fasting and postprandial state. The *I* ^2^ statistic was over 80% for all GI hormones, both in the fasting and postprandial states, except for active and total GLP‐1 with *I* ^2^ statistic of 32.8 and 62.4%, respectively (Table 1). Forrest plots for meta‐analyses of basal concentrations of GI hormones are presented in Figure 2A–G and postprandial concentrations (AUC) in Figure 3A–C. In the comparison of basal concentrations, the pooled SMDs were observed to be lower in obesity for basal active and total ghrelin, total GLP‐1, active PYY, and total PYY, although this was only statistically significant for basal total ghrelin (SMD: −1.42, 95% CI −2.07 to −0.76, *I* ^2^ = 96.96%, *p* < 0.001) (Figure 2B). In the comparison of AUC, the pooled SMDs for total ghrelin, active GLP‐1 and total PYY also indicated lower postprandial concentrations in the obesity group, with the AUCs for both total ghrelin and total PYY being statistically significantly smaller in obesity (Figure 3A and Figure 3C, respectively). For the other GI hormones, the pooled SMD was associated with a large degree of uncertainty, such that it is not possible to conclude whether these hormones differ been individuals with and without obesity. In particular, the confidence intervals for basal active ghrelin, active GLP‐1, total GLP‐1, active PYY, total PYY, and CKK were consistent with there being between a substantially lower concentration in obesity, no difference, or even a moderately to substantially higher concentration in obesity. In contrast, the pooled results from the seven studies reporting on basal active GLP‐1 indicated that any difference between the obesity and control group is likely to be small (SMD: 0.14, 95% CI −0.12 to 0.40, *p* = 0.29, *I* ^2^ = 32.8%).

Visually, the funnel plots for active‐ and total ghrelin and total GLP‐1 are suggestive of some publication bias, with the smaller published studies reporting large effect sizes. Furthermore, Egger's test also implied that there may be a small study effects for total ghrelin and total GLP‐1 **(**Table 1). The funnel plots and Egger's test for other GI hormones, in the fasting and postprandial states, should be interpreted cautiously given that there are fewer studies in these comparisons.

#### 3.3.2 Appetite ratings in the fasting and postprandial state

**FIGURE 4:**
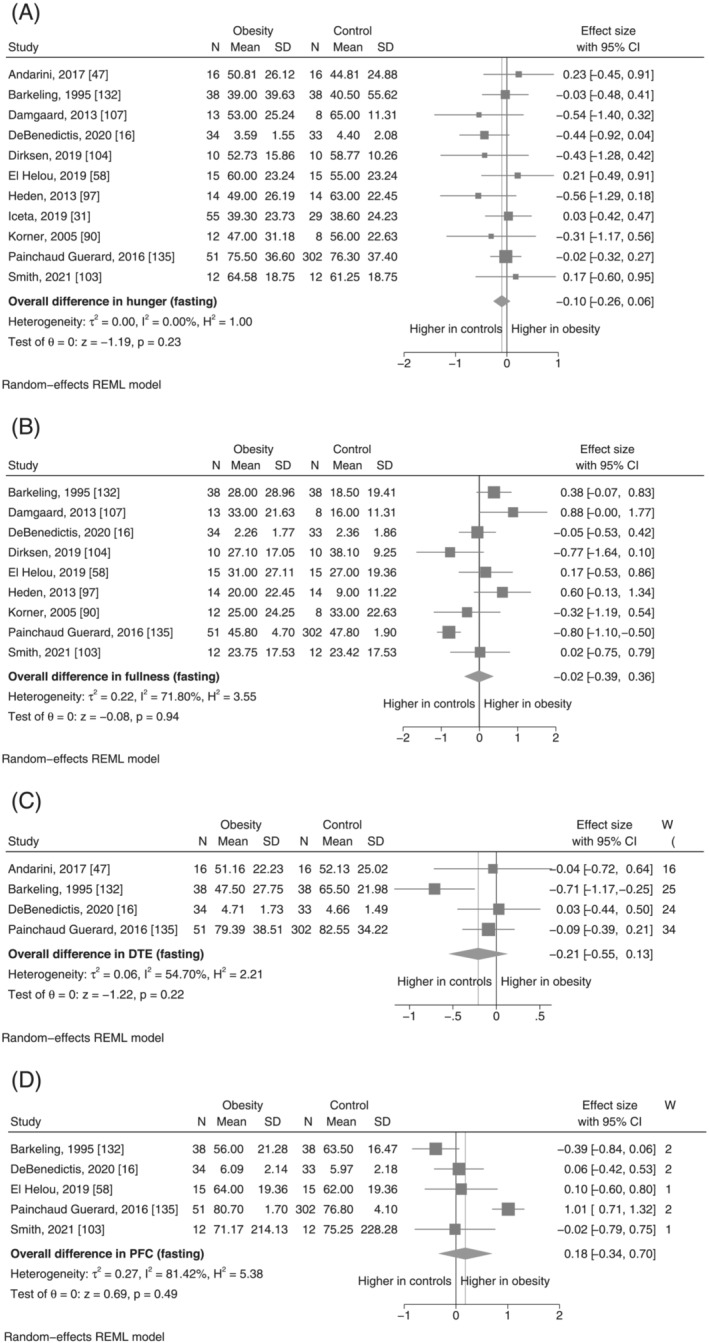
(A–D) Meta‐analysis results for fasting appetite ratings

**FIGURE 5:**
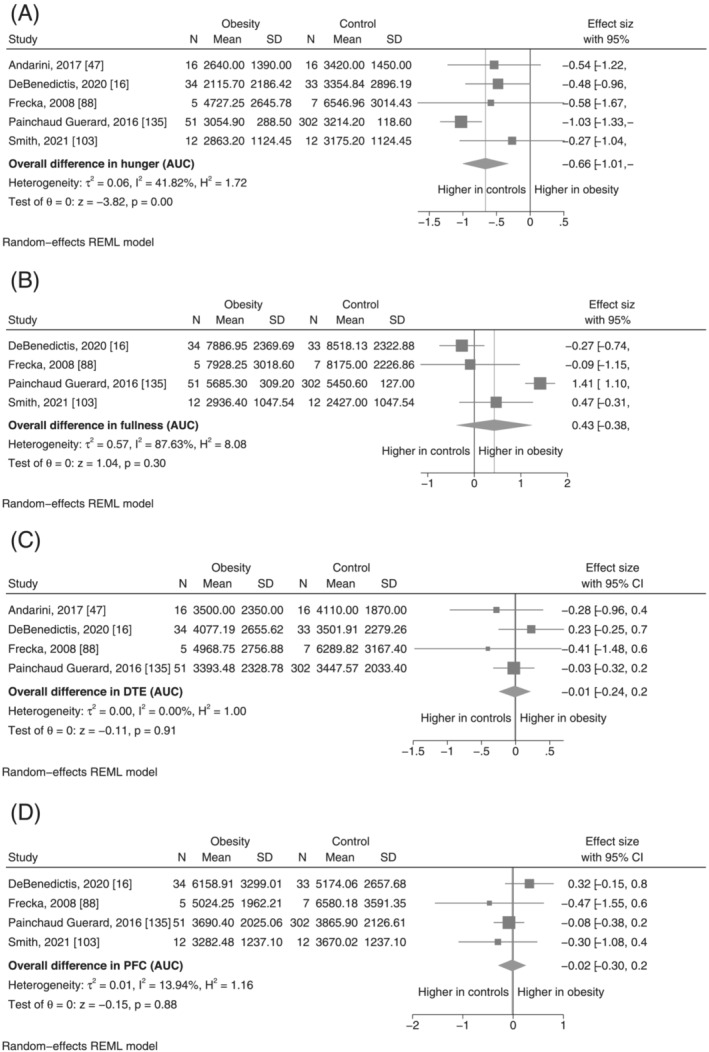
(A–D) Meta‐analysis results for postprandial appetite ratings

Compared with the GI hormone concentrations, there was less statistical heterogeneity in the comparison of appetite ratings, although still high for fullness, both in the fasting and postprandial state, and fasting PFC (Table 1). The pooled SMD for fasting appetite ratings was small and not statistically significant (Figure 4A–D). In the comparison of postprandial hunger, the pooled SMD indicated lower hunger AUC in the obesity group (SMD: −0.66, 95% CI −1.01 to −0.32, *p* < 0.001, *I* ^2^ = 41.8) (Figure 5A). Postprandial fullness was observed to be higher in obesity, although this was associated with a high degree of statistical uncertainty and heterogeneity, and was not statistically significant (SMD: 0.43, 95% −0.38 to 1.24, *p* = 0.30, *I* ^2^ = 87.6%) (Figure 5B). The estimated SMD and associated confidence intervals for meta‐analyses of postprandial DTE and PFC indicate that it is unlikely to be any substantial difference in these appetite feelings between individuals with obesity and controls (Figure 5C and Figure 5D, respectively).

## 4 DISCUSSION

This systematic review and meta‐analysis represent the first comprehensive effort to investigate if the plasma concentration of GI hormones and subjective appetite ratings differ between adults with, and without, obesity. The systematic review showed a trend toward an attenuated hormonal response to nutrient ingestion in individuals with obesity. The meta‐analysis showed that individuals with obesity indeed present with statistical significantly lower basal and postprandial total ghrelin concentrations compared with controls, lower postprandial concentrations of total PYY, and lower postprandial hunger ratings. No convincing differences were found for GLP‐1, CCK, or fullness, DTE or PFC ratings. However, there was a large methodological and statistical heterogeneity among studies. Interestingly, a similar systematic review and meta‐analysis previously conducted in children reported the same results, with an attenuated postprandial total ghrelin and total PYY response in children with obesity, despite large heterogeneity.^137^

Ghrelin is produced in the fundus of the stomach and stimulates appetite. Its concentration peaks during fasting, and in anticipation of the upcoming meal, and declines in the postprandial period.^8^, ^9^ It has long been reported that individuals with obesity have a lower basal and postprandial concentration of ghrelin compared with controls.^28^ This meta‐analysis confirmed these findings. However, for active ghrelin, the differences were associated with a large degree of uncertainty, preventing conclusions to be drawn.

As macronutrients interact with receptors in enteroendocrine cells, satiety peptides are secreted from the GI tract.^10^, ^11^, ^12^, ^138^ It is generally accepted that obesity is associated with lower postprandial concentrations of satiety peptides and a weaker satiation/satiety.^13^, ^14^, ^94^, ^114^, ^139^ Centrally, through hypothalamic actions and the vagal‐brainstem signaling pathway, GLP‐1 promotes satiation and post meal satiety by reducing food intake in a dose‐dependent manner.^140^, ^141^ The present meta‐analysis, however, found no conclusive evidence of a difference in the plasma concentration of active or total GLP‐1 between individuals with and without obesity. There was an overall trend for a higher postprandial concentration of active GLP‐1 in controls, but this was not statistically significant, and with a high degree of statistical heterogeneity among the studies. Discrepancies in the literature have previously been observed in both human and animal studies.^142^ And as such, it may be difficult to determine the role of GLP‐1 in common obesity, especially in a state of weight stability.

PYY is thought to be involved in post meal satiety^143^ and thereby decrease food intake.^12^ Its plasma concentrations increase within 15–30 min after a meal and peak around 60–90 min postprandially. The quantitative analysis demonstrated that postprandial total PYY concentrations were statistically significantly lower in individuals with obesity compared with controls. This is in line with a previously published paper from our group showing that PYY postprandial response is poor in class I obesity compared with individuals without obesity, and completely absent in class II and III obesity.^13^ It has been suggested that the lower postprandial PYY concentrations measured in obesity would result in increased food intake in order to achieve the same degree of fullness as that seen normal weight individuals.^139^

CCK is the best established and most important satiation signal, being involved in meal termination, and possibly also early phase satiety,^144^, ^145^, ^146^, ^147^ and acts primarily through vagal afferent fibers.^148^, ^149^ Unfortunately, few studies have assessed concentrations of CCK in individuals with and without obesity, and we were therefore unable to run a meta‐analysis on this hormone. Based on the systematic review alone, results were rather inconclusive. Even if no true differences in CCK (or GLP‐1) plasma concentrations exist between individuals with and without obesity, one cannot rule out the importance of the vagus nerve in regulating appetite. The chronic ingestion of energy‐rich diets has been shown to reduce the sensitivity of vagal afferent neurons to peripheral signals, which would be sufficient to drive both hyperphagia and obesity.^150^

In laboratorial settings, infusion of GI hormones has been shown to affect eating behavior in both individuals with normal‐weight and obesity.^12^, ^86^, ^138^, ^139^, ^151^ However, the association between plasma concentration of GI hormones and appetite ratings is highly complex and the evidence for their influence on food intake at normal physiological levels less clear.^152^ An important aspect to consider is that subjective appetite ratings do not provide the full spectrum of either appetite control/behaviors or actual food intake. Subjective appetite ratings may merely represent an individual's interpretation of his/her feelings and motivations to eat, rather than direct measures of the underlying physiological processes controlling eating.^40^, ^153^, ^154^ It was early reported that subjective appetite ratings in individuals with and without obesity showed similar sensitivity to macronutrients^155^ and dietary manipulations.^156^ However, because obesity is associated with greater energy intake,^157^ it was somewhat surprising that in the present meta‐analysis, individuals with obesity presented with lower postprandial hunger ratings compared with controls. One hypothesis is that obesity, through distinctive processes (i.e., reduced vagal sensitivity as previously discussed, or other central mechanisms), dysregulates the sensing of appetite. For example, in individuals with a normal weight, ghrelin and PYY concentrations are correlated with hunger and fullness ratings, respectively, whereas no association has been found in individuals with obesity.^158^ A second alternative hypothesis is that once an individual reaches his/her genetically determined weight, no differences in appetite markers are seen between individuals with normal‐weight and obesity. However, the lower ghrelin plasma concentrations, both in the fasting and postprandial states, and lower postprandially PYY secretion seen in individuals with obesity, questions this hypothesis and asks for more research. Finally, it is also possible that food intake in individuals with obesity is driven by the hedonic system. There is evidence that GI hormones also mediate the hedonic appetite system.^159^ Hedonic appetite can easily override homeostatic signals when food is easily available and highly palatable, even in the absent of physiological hunger.^160^ This is particularly important considering the obesogenic environment that has emerged over the past decades. Hedonic characteristics might, therefore, offer an additional proxy to help reflect motivation to eat and actual food intake.

Diet‐induced weight loss has consistently been shown to modulate the plasma concentration of GI hormones, and appetite ratings, including increases in ghrelin plasma concentrations, as well as hunger and fullness ratings,^16^, ^161^, ^162^ despite inconsistent findings regarding satiety peptides.^14^, ^16^, ^18^ Interestingly, our research group has recently demonstrated that the increased orexigenic drive to eat seen after weight loss likely reflects a normalization toward a lower body weight, given that no differences were seen between reduced‐obese individuals and fat mass matched controls.^16^ Moreover, maintaining weight loss after dietary restriction has been suggested to be dependent on increases in postprandial GLP‐1 and PYY responses.^163^ Even though establishing a direction of causality if difficult, and out of the scope of this review, the previously discussed findings point to obesity being a cause, not a consequence, of potential abnormalities in the homeostatic appetite control system.

This review has several strengths. It is the first systematic review and meta‐analysis comparing GI hormones and appetite ratings between adults with obesity and controls. Second, the analysis was conducted following the PRISMA statement guidelines, and used well established tools during the whole selection process. Third, its comprehensiveness provides a warranted descriptive picture of the topic of interest. Unfortunately, this analysis also has some limitations. First, several studies were not included in the meta‐analysis due to missing data. This might have affected the results and contributed to the lack of significant differences between groups for some of the outcome variables. Second, the heterogeneity in most meta‐analyses conducted in this study was also relatively high and, as such, conclusions should be made with caution. When interpreting the present results, it is critical to consider timing, nature, and structure of the test‐meal, important aspects in modulating the outcome variables of this review. The statistical heterogeneity seen in this meta‐analysis, particularly for the GI hormone comparisons, is likely to be the result of differences in the underlying study populations, test‐meals used, and sample processing. For example, GLP‐1 is rapidly degraded, and accurate methods are crucial to obtain precise measures. Also, the adequate measure of L‐cell secretion is total GLP‐1, whereas active GLP‐1 provides information about the endocrine part of the peptide's actions.^164^ Thus, developing an optimized standardized method to assess hormonal responses and subjective appetite is needed. Third, transparent research and reporting of results should be encouraged, as there is some indication of publication bias with an apparent small study effect among the published results for basal hormone concentration. Fourth, different GI hormones are stimulated by specific nutrients in the lumen.^5^ This means that the mixed meals used (liquid or solid), with different macronutrient composition, or even single macronutrient meals, might not have been the best to maximize the inhibition of ghrelin, or the release of the different satiety peptides. For example, among the included studies in the analysis for active GLP‐1 (AUC), higher concentrations were seen in controls, versus individuals with obesity, after a 750 kcal liquid meal,^15^ whereas no differences were reported between groups after a 450–650 kcal solid meal.^16^, ^94^, ^97^ Another important aspect to take into consideration is if the tests meals were similar in the obesity and control groups, or if they were adjusted for body weight. Most of the studies have used the same test meal in both groups. This ensures that the same stimuli is given to each subject but does not account for individual nutritional needs. Lastly, although all efforts were made to ensure that measures were comparable, the length of the postprandial period could affect the results.

## 5 CONCLUSION

Obesity is associated with lower basal and postprandial concentrations of total ghrelin, lower postprandial concentration of total PYY, and lower postprandial hunger ratings, but large variations exist. More studies are needed to better understand the implications of these findings and to determine if they are a cause or a consequence of obesity. Further, it is important to establish if an association exists between the alterations in GI hormones seen in individuals with obesity and their actual food intake. This will provide a better understanding of the pathophysiology of this chronic disease.

## CONFLICT OF INTEREST

The authors have no conflict of interest to disclosure.

## AUTHOR CONTRIBUTIONS

Catia Martins, Marthe Isaksen Aukan, and Silvia Coutinho formulated the research questions and designed the study. [Correction added on 15 December 2022, after first online publication: The author names have been updated in the preceding sentence, in this version.] Marthe Isaksen Aukan and Silvia Coutinho conducted the reviewer process. Sindre Andre Pedersen conducted the literature search. Melanie Rae Simpson conducted the statistical analysis. All authors were involved in the writing of the manuscript.

## Supporting information


**Data S1** Supporting Information
