# Supplementary material for: Differences in gastrointestinal hormones and appetite ratings between individuals with and without obesity—A systematic review and meta‐analysis
Source: Obes Rev. 2022 Nov 23;24(2):e13531. doi: 10.1111/obr.13531 (PMC10078575; doi:10.1111/obr.13531)
Supplement: Supplementary file 1 — Data S1 Supporting Information [file OBR-24-0-s001.pdf]

Supplementary file

Differences in gastrointestinal hormones and appetite ratings between individuals with and without obesity – A systematic review and meta-analysis

Authors: Marthe Isaksen Aukan [1,2], Silvia Coutinho [1,3], Sindre Andre Pedersen [4], Melanie Rae Simpson[5,6] and Catia Martins [1,2,7]

[1] Obesity Research Group, Department of Clinical and Molecular Medicine, Faculty of Medicine, Norwegian University of Science and Technology (NTNU), Trondheim, Norway; [2] Centre of Obesity and Innovation (ObeCe), Clinic of Surgery, St. Olav University Hospital, Trondheim, Norway; [3] Department of Public Health Nutrition at the Institute of Basic Medical Sciences, Faculty of Medicine, University of Oslo (UiO), Norway; [4] Library Section for Research support, Data and Analysis, Norwegian University of Science and Technology (NTNU), Trondheim, Norway [5]Department of Public Health and Nursing, Norwegian University of Science and Technology, Trondheim, Norway, [6]Clinical Research Unit Central Norway, St. Olavs Hospital, Trondheim, Norway, [7] Department of Nutrition Sciences, the University of Alabama at Birmingham (UAB), Birmingham, AL, USA.

Contents

Search strategy: 3
- MEDLINE 3
- Embase 5
- PsycINFO 7
- Cochrane Library 8
- Web of Science 10
- ClinicalTrials.gov 10

Flow diagram 12

Summary of contributing articles 13
- Active ghrelin (basal) 13
- Total ghrelin (basal) 15
- Active GLP-1 (basal) 17
- Total GLP-1 (basal) 19
- Active PYY (basal) 21
- Total PYY (basal) 23
- CKK (basal) 25
- Total ghrelin (AUC) 27
- Active GLP-1 (AUC) 29
- Total PYY (AUC) 31
- Hunger (fasting) 33
- Fullness (fasting) 35
- Desire to eat (DTE) (fasting) 37
- Prospective food consumption (PFC) (fasting) 39
- Hunger (AUC) 41
- Fullness (AUC) 43
- Desire to eat (DTE) (AUC) 45
- Prospective food consumption (PFC) (AUC) 47

Supplementary table 19 – Risk of bias assessment 49

Supplementary table 20. Study characteristics 54

Search strategy:

MEDLINE

Ovid MEDLINE(R) ALL <1946 to September 01, 2021>

#	**Searches**
1	exp Obesity/
2	exp Obesity, Morbid/
3	exp Obesity, Abdominal/
4	exp Overweight/
5	adipos*.ti,ab,kw.
6	obes*.ti,ab,kw.
7	overweight*.ti,ab,kw.
8	over-weight*.ti,ab,kw.
9	((high* or unhealthy) adj3 (body-mass-ind* or BMI?)).ti,ab,kw.
10	or/1-9 [Combined thesaurus and text words for overweight/obesity concept]
11	exp Ideal Body Weight/
12	normal-weight*.ti,ab,kw.
13	normal-body-weight*.ti,ab,kw.
14	normal-body-mass*.ti,ab,kw.
15	healthy-weight*.ti,ab,kw.
16	healthy-body-weight*.ti,ab,kw.
17	healthy-body-mass*.ti,ab,kw.
18	non-obese.ti,ab,kw.
19	nonobese.ti,ab,kw.
20	non-overweight*.ti,ab,kw.
21	slim.ti,ab,kw.
22	lean.ti,ab,kw.
23	((normal or control? or healthy or low*) adj3 (overweight or over-weight or obese* or men or women or subject* or person* or body-mass-ind* or BMI? or volunteer*)).ti,ab,kw.
24	or/11-23 [Combined thesaurus and text words for healthy weight concept]
25	exp Hunger/
26	exp Appetite/
27	exp Appetite Regulation/
28	exp Satiation/
29	appetite?.ti,ab,kw.
30	apetite?.ti,ab,kw.
31	hunger.ti,ab,kw.

32 fullness.ti,ab,kw.

33 satiation.ti,ab,kw.

34 satiety.ti,ab,kw.

35 prospective-food-consumption*.ti,ab,kw.

36 ((desire or craving*) adj3 (eat* or food or feed*)).ti,ab,kw.

37 exp Ghrelin/

38 ghrelin.ti,ab,kw.

39 GHRL.ti,ab,kw.

40 appetite-regulating-hormone.ti,ab,kw.

41 exp Cholecystokinin/

42 cholecystokinin.ti,ab,kw.

43 CCK.ti,ab,kw.

44 uropancreozymin.ti,ab,kw.

45 pancreozymin.ti,ab,kw.

46 exp Peptide-YY/

47 peptide-YY.ti,ab,kw.

48 PYY.ti,ab,kw.

49 PYY3-36.ti,ab,kw.

50 peptide-tyrosine-tyrosine.ti,ab,kw.

51 exp Glucagon-Like Peptide 1/

52 glucagon-like-peptide-1.ti,ab,kw.

53 GLP-1.ti,ab,kw.

54 GLP1.ti,ab,kw.

55 or/25-54 [Combined thesaurus and text words for appetite/appetite markers concept]

56 and/10,24,55

57 56 not (comment or editorial or news or newspaper article).pt. [Excluded publication types]

58 (Animals/ or Models, Animal/ or Disease Models, Animal/) not Humans/

59 ((animal or animals or canine* or dog or dogs or feline or hamster* or lamb or lambs or mice or monkey or monkeys or mouse or murine or pig or pigs or piglet* or porcine or primate* or rabbit* or rat or rats or rodent* or sheep* or veterinar*) not (human* or patient*)).ti,kf,jw.

60 57 not (58 or 59) [Animal-only studies excluded]

61 (exp infant/ or exp child/ or exp adolescent/) not exp adult/

62 ((child* or stepchild* or step-child* or kid or kids or girl or girls or boy or boys or teen* or youth* or youngster* or adolescent* or adolescence or preschool* or pre-school* or kindergarten* or school* or juvenile* or minors or p?ediatric* or PICU) not adult*).ti,ab.

63 60 not (61 or 62) [Non-adults only studies excluded]

64 limit 63 to (english or norwegian or swedish or danish or portuguese or french or spanish) [Limiting results to English, Norwegian, Swedish, Danish, Portugese, French or Spanish language]

Embase

Embase <1974 to 2021 September 01>

#	Searches
1	exp Obesity/
2	exp Morbid Obesity/
3	exp Abdominal Obesity/
4	exp Obesity Management/
5	exp Maternal Obesity/
6	adipos*.ti,ab,kw.
7	obes*.ti,ab,kw.
8	overweight*.ti,ab,kw.
9	over-weight*.ti,ab,kw.
10	((high* or unhealthy) adj3 (body-mass-ind* or BMI?)).ti,ab,kw.
11	or/1-10 [Combined thesaurus and text words for overweight/obesity concept]
12	exp Ideal Body Weight/
13	normal-weight*.ti,ab,kw.
14	normal-body-weight*.ti,ab,kw.
15	normal-body-mass*.ti,ab,kw.
16	healthy-weight*.ti,ab,kw.
17	healthy-body-weight*.ti,ab,kw.
18	healthy-body-mass*.ti,ab,kw.
19	non-obese.ti,ab,kw.
20	nonobese.ti,ab,kw.
21	non-overweight*.ti,ab,kw.
22	slim.ti,ab,kw.
23	lean.ti,ab,kw.
24	((normal or control? or healthy or low*) adj3 (overweight or over-weight or obese* or men or women or subject* or person* or body-mass-ind* or BMI? or volunteer*)).ti,ab,kw.
25	or/12-24
26	or/12-24 [Combined thesaurus and text words for healthy weight concept]
27	exp Hunger/
28	exp Appetite/
29	exp Decreased Appetite/
30	exp "Loss Of Appetite"/
31	exp Appetite Disorder/
32	exp Appetite Stimulant/
33	exp Increased Appetite/
34	exp Satiety/
35	appetite?.ti,ab,kw.

36	apetite?.ti,ab,kw.
37	hunger.ti,ab,kw.
38	fullness.ti,ab,kw.
39	satiation.ti,ab,kw.
40	satiety.ti,ab,kw.
41	prospective-food-consumption*.ti,ab,kw.
42	((desire or craving*) adj3 (eat* or food or feed*)).ti,ab,kw.
43	exp Ghrelin/
44	ghrelin.ti,ab,kw.
45	GHRL.ti,ab,kw.
46	appetite-regulating-hormone.ti,ab,kw.
47	exp Cholecystokinin/
48	cholecystokinin.ti,ab,kw.
49	CCK.ti,ab,kw.
50	uropancreozymin.ti,ab,kw.
51	pancreozymin.ti,ab,kw.
52	exp Peptide-YY/
53	peptide-YY.ti,ab,kw.
54	PYY.ti,ab,kw.
55	PYY3-36.ti,ab,kw.
56	peptide-tyrosine-tyrosine.ti,ab,kw.
57	exp Glucagon-Like Peptide 1/
58	glucagon-like-peptide-1.ti,ab,kw.
59	GLP-1.ti,ab,kw.
60	GLP1.ti,ab,kw.
61	or/27-60 [Combined Combined thesaurus and text words for appetite/appetite markers concept]
62	and/11,26,61
63	62 not (comment or editorial or news or newspaper article).pt. [Exluded publication types]
64	(exp Animal/ or exp Juvenile Animal/ or Adult Animal/ or Animal Cell/ or Animal Tissue/ or Nonhuman/ or Animal Experiment/ or Animal Model/) not Human/
65	(animal or animals or canine* or dog or dogs or feline or hamster* or lamb or lambs or mice or monkey or monkeys or mouse or murine or pig or pigs or piglet* or porcine or primate* or rabbit* or rat or rats or rodent* or sheep* or veterinar*).ti,kw,dq,jx. not (human* or patient*).mp.
66	63 not (64 or 65) [Animal-only studies excluded]
67	(exp Infant/ or exp Child/ or exp Adolescent/) not exp Adult/
68	((child* or stepchild* or step-child* or kid or kids or girl or girls or boy or boys or teen* or youth* or youngster* or adolescent* or adolescence or preschool* or pre-school* or kindergarten* or school* or juvenile* or minors or p?ediatric* or PICU) not adult*).ti,ab.
69	66 not (67 or 68) [non-adults only studies excluded]

70	limit 69 to (english or norwegian or swedish or danish or portuguese or french or spanish) [Limiting results to English, Norwegian, Swedish, Danish, Portugese, French or Spanish language]
71	limit 70 to medline
72	70 not (medline or "pubmed not medline").ns. [Excluding MEDLINE and PubMed not MEDLINE records]

PsycINFO

APA PsycInfo <1806 to August Week 4 2021>

#	Searches
1	exp Obesity/
2	exp Overweight/
3	adipos*.tw.
4	obes*.tw.
5	overweight*.tw.
6	over-weight*.tw.
7	((high* or unhealthy) adj3 (body-mass-ind* or BMI?)).tw.
8	or/1-7 [Combined thesaurus and text words for overweight/obesity concept]
9	normal-weight*.tw.
10	normal-body-weight*.tw.
11	normal-body-mass*.tw.
12	healthy-weight*.tw.
13	healthy-body-weight*.tw.
14	healthy-body-mass*.tw.
15	non-obese.tw.
16	nonobese.tw.
17	non-overweight*.tw.
18	slim.tw.
19	lean.tw.
20	((normal or control? or healthy or low*) adj3 (overweight or over-weight or obese* or men or women or subject* or person* or body-mass-ind* or BMI? or volunteer*)).tw.
21	or/9-20 [Combined thesaurus and text words for healthy weight concept]
22	exp Hunger/
23	exp Appetite/
24	exp Satiation/
25	appetite?.tw.
26	apetite?.tw.
27	hunger.tw.
28	fullness.tw.

29	satiation.tw.
30	satiety.tw.
31	prospective-food-consumption*.tw.
32	((desire or craving*) adj3 (eat* or food or feed*)).tw.
33	exp Ghrelin/
34	ghrelin.tw.
35	GHRL.tw.
36	appetite-regulating-hormone.tw.
37	exp Cholecystokinin/
38	cholecystokinin.tw.
39	CCK.tw.
40	uropancreozymin.tw.
41	pancreozymin.tw.
42	peptide-YY.tw.
43	PYY.tw.
44	PYY3-36.tw.
45	peptide-tyrosine-tyrosine.tw.
46	glucagon-like-peptide-1.tw.
47	GLP-1.tw.
48	GLP1.tw.
49	or/22-48 [Combined thesaurus and text words for appetite/appetite markers]
50	and/8,21,49
51	50 not ("column opinion" or "comment reply" or "Editorial").dt. [Excluded document types]
52	((human or animal) not human).po.
53	(animal or animals or canine* or dog or dogs or feline or hamster* or lamb or lambs or mice or monkey or monkeys or mouse or murine or pig or pigs or piglet* or porcine or primate* or rabbit* or rat or rats or rodent* or sheep* or veterinar*).ti,ab. not (human* or patient*).mp.
54	51 not (52 or 53) [Animal-only studies excluded]
55	((adolescence 13 17 yrs or adulthood 18 yrs older or childhood birth 12 yrs) not adulthood 18 yrs older).ag.
56	((child* or stepchild* or step-child* or kid or kids or girl or girls or boy or boys or teen* or youth* or youngster* or adolescent* or adolescence or preschool* or pre-school* or kindergarten* or school* or juvenile* or minors or p?ediatric* or PICU) not adult*).ti,ab.
57	54 not (55 or 56) [non-adult studies excluded]
58	limit 57 to (english or norwegian or swedish or danish or portuguese or french or spanish) [Limiting results to English, Norwegian, Swedish, Danish, Portugese, French or Spanish language]

Cochrane Library

ID Search Hits

#1 (adipos*):ti,ab,kw
#2 (obes*):ti,ab,kw
#3 (overweight*):ti,ab,kw
#4 (over-weight*):ti,ab,kw
#5 ((high* or unhealthy) NEAR/3 (body-mass-ind* or BMI?)):ti,ab,kw
#6 #1 OR #2 OR #3 OR #4 OR #5
#7 (normal-weight*):ti,ab,kw
#8 (normal-body-weight*):ti,ab,kw
#9 (normal-body-mass*):ti,ab,kw
#10 (healthy-weight*):ti,ab,kw
#11 (healthy-body-weight*):ti,ab,kw
#12 (healthy-body-mass*):ti,ab,kw
#13 (non-obese):ti,ab,kw
#14 (nonobese):ti,ab,kw
#15 (non-overweight*):ti,ab,kw
#16 (slim):ti,ab,kw
#17 (lean):ti,ab,kw
#18 ((normal or control? or healthy or low*) NEAR/3 (overweight or over-weight or obese* or men or women or subject* or person* or body-mass-ind* or BMI? or volunteer*)):ti,ab,kw
#19 #7 OR #8 OR #9 OR #10 OR #11 OR #12 OR #13 OR #14 OR #15 OR #16 OR #17 OR #18
#20 (appetite?):ti,ab,kw
#21 (apetite?):ti,ab,kw
#22 (hunger):ti,ab,kw
#23 (fullness):ti,ab,kw
#24 (satiation):ti,ab,kw
#25 (satiety):ti,ab,kw
#26 (prospective-food-consumption*):ti,ab,kw
#27 ((desire or craving*) NEAR/3 (eat* or food or feed*)):ti,ab,kw
#28 (ghrelin):ti,ab,kw
#29 (GHRL):ti,ab,kw
#30 (appetite-regulating-hormone):ti,ab,kw
#31 (cholecystokinin):ti,ab,kw
#32 (CCK):ti,ab,kw
#33 (uropancreozymin):ti,ab,kw
#34 (pancreozymin):ti,ab,kw
#35 (peptide-YY):ti,ab,kw
#36 (PYY):ti,ab,kw
#37 ("PYY3-36"):ti,ab,kw
#38 (peptide-tyrosine-tyrosine):ti,ab,kw
#39 (glucagon-like-peptide-1):ti,ab,kw
#40 (GLP-1):ti,ab,kw
#41 (GLP1):ti,ab,kw
#42 #20 OR #21 OR #22 OR #23 OR #24 OR #25 OR #26 OR #27 OR #28 OR #29 OR #30 OR #31 OR #32 OR #33 OR #34 OR #35 OR #36 OR #37 OR #38 OR #39 OR #40 OR #41
#43 #6 AND #19 AND #42
#44 ((child* OR stepchild* OR step-child* OR kid OR kids OR girl OR girls OR boy OR boys OR teen* OR youth* OR youngster* OR adolescent* OR adolescence OR preschool* OR pre-school* OR kindergarten* OR school* OR juvenile* OR minors OR p?ediatric* OR PICU) NOT adult*):ti,ab,kw

#45 #43 NOT #44

Web of Science

Web of Science Core Collection

16	#13 not #14 and Editorial Materials (Exclude – Document Types)
15	#13 not #14
14	TS=(("child*" or "stepchild*" or "step-child*" or "kid" or "kids" or "girl" or "girls" or "boy" or "boys" or "teen*" or "youth*" or "youngster*" or "adolescent*" or "adolescence" or "preschool*" or "pre-school*" or "kindergarten*" or "school*" or "juvenile*" or "minors" or "p?ediatric*" or "PICU") not "adult*")
13	#11 not #12
12	TS=(("animal" or "animals" or "canine*" or "dog" or "dogs" or "feline" or "hamster*" or "lamb" or "lambs" or "mice" or "monkey" or "monkeys" or "mouse" or "murine" or "pig" or "pigs" or "piglet*" or "porcine" or "primate*" or "rabbit*" or "rat" or "rats" or "rodent*" or "sheep*" or "veterinar*") not ("human*" or "patient*"))
11	#10 AND #6 AND #3
10	#9 OR #8 OR #7
9	TS=("ghrelin" or "GHRL" or "appetite-regulating-hormone" or "cholecystokinin" or "CCK" or "uropancreozymin" or "pancreozymin" or "peptide-YY" or "PYY" or "PYY3-36" or "peptide-tyrosine-tyrosine" or "glucagon-like-peptide-1" or "GLP-1" or "GLP1")
8	TS=(("desire" or "craving*") NEAR/3 ("eat*" or "food" or "feed*"))
7	TS=("appetite?" or "apetite?" or "hunger" or "fullness" or "satiation" or "satiety" or "prospective-food-consumption*")
6	#4 OR #5
5	TS=((normal or control? or healthy or low*) NEAR/3 (overweight or over-weight or obese* or men or women or subject* or person* or body-mass-ind* or BMI? or volunteer*))
4	TS=("normal-weight*" or "normal-body-weight*" or "normal-body-mass*" or "healthy-weight*" or "healthy-body-weight*" or "healthy-body-mass*" or "non-obese" or "nonobese" or "non-overweight*" or "slim" or "lean")
3	#2 OR #1
2	TS=(("high*" or "unhealthy") NEAR/3 ("body-mass-ind*" or "BMI?"))
1	TS=("adipos*" or "obes*" or "overweight*" or "over-weight*")

ClinicalTrials.gov

(hunger OR appetite OR fullness OR satiation OR satiety OR prospective-food-consumption OR desire OR craving OR cravings OR ghrelin OR GHRL OR appetite-regulating-hormone OR cholecystokinin OR PYY OR PYY3-36 OR GLP-1 OR GLP1) AND (obese OR obesity OR overweight OR over-weight OR body-mass-index OR BMI OR overweight) AND (normal-weight OR normal-body-weight OR normal-body-mass OR healthy-weight OR healthy-body-weight OR healthy-body-mass OR non-obese OR nonobese OR lean OR slim) AND (Adult OR Older Adult)

Flow diagram

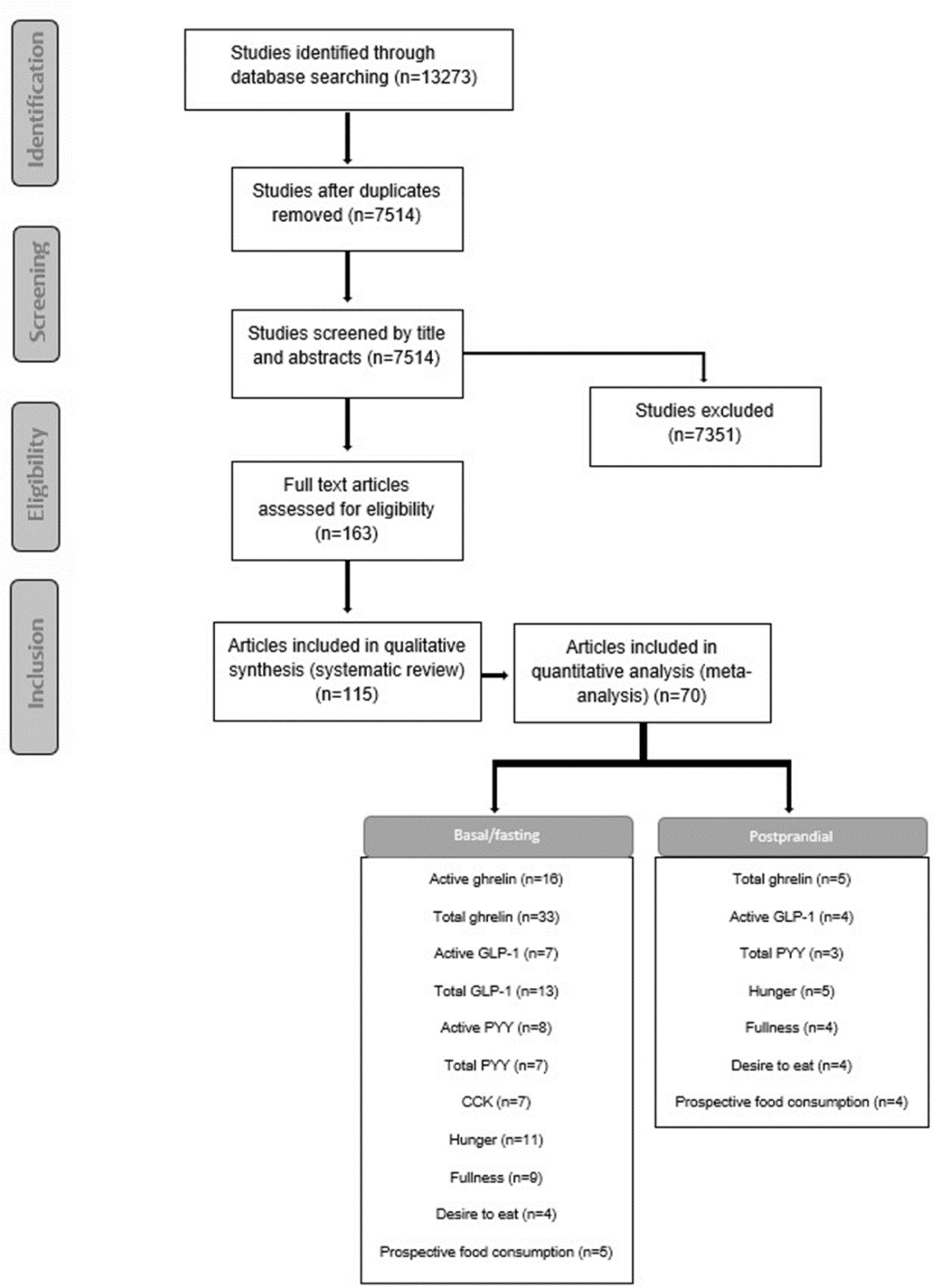

Figure 1: Flow diagram of the selection process.

Summary of contributing articles

Basal hormone concentrations

Active ghrelin (basal)

Table 1: Summary of contributing articles for active ghrelin (basal)

		Obesity group				Control group			
**Author (year)**	**Study design**	**n**	**% female**	**Age (years)**	**BMI (kg/m²)**	**n**	**% female**	**Age (years)**	**BMI (kg/m²)**
Andarini, 2017 [47]	Cross-sectional	16	0.0	21.4 (1.9)	33.6 (4.8)	16	0.0	20.6 (1.1)	21.3 (1.1)
Arafat, 2013 [26]	Parallel RCT	11	55.0	28.4 (8.6)	34.4 (5.6)	13	54.0	25.1 (2.2)	21.7 (2.2)
Carrol, 2007 [40]	Cross-sectional	20	55.0	47.2 (11.0)	36.8 (4.8)	19	47.0	39.2 (10.3)	22.6 (1.2)
Chearskul, 2012 [41]	Cross-sectional	33	100.0	33.6 (6.3)	29.4 (3.6)	53	100.0	30.1 (5.8)	20.7 (2.2)
Dardzinska, 2014 [27]	.	12	71.0	35.4 (9.1)	43.8 (6.8)	12	92.0	37.2 (9.4)	23.0 (3.5)
DeBenedictis, 2020 [16]	Cross-sectional	34	50.0	38.9 (2.0)	34.0 (0.4)	33	49.0	45.0 (1.5)	24.8 (0.4)
Douglas, 2017 [42]	Crossover	25	50.0	45.0 (12.4)	29.2 (2.9)	22	44.0	37.5 (15.2)	22.4 (1.5)
Heden, 2013 [97]	Crossover	14	57.0	25.1 (5.0)	34.8 (4.4)	14	43.0	26.0 (6.0)	22.9 (1.7)
Homaee, 2011 [30]	Cross-sectional	19	0.0	27.5 (5.8)	31.0 (3.6)	19	0.0	26.9 (5.6)	18.5 (2.2)
Iceta, 2019 [31]	Cross-sectional	55	100.0	38.0 (11.1)	41.5 (5.9)	29	100.0	37.0 (10.8)	21.5 (2.2)
Karcz-Socha, 2011 [45]	Cross-sectional	96	50.0	51.5 (6.5)	35.3 (2.9)	46	52.0	51.2 (6.5)	23.4 (1.5)
Kolodziejski, 2018 [43]	Cross-sectional	15	100.0	53.0 (8.5)	39.8 (1.0)	15	100.0	58.6 (10.3)	22.3 (0.5)
Korek, 2013 [32]	Cross-sectional	19	90.0	42.2 (3.3)	34.7 (4.9)	17	89.0	42.9 (5.3)	21.1 (1.9)
Krzyzanowska-Swinirska, 2007 [46]	Cross-sectional	30	100.0	44.3 (4.3)	34.4 (4.1)	32	100.0	30.6 (3.6)	21.3 (1.7)
Lopez-Aguilar, 2018 [33]	Cross-sectional	50	52.0	29.2 (6.3)	34.5 (5.2)	80	67.0	26.4 (5.6)	22.7 (1.5)
Marzullo, 2004 [35]	Cross-sectional	20	50.0	31.8 (2.5)	41.3 (1.1)	20	50.0	33.5 (2.4)	22.4 (0.6)

BMI: Body mass index; NR: not reported; Values for age and BMI are presented as mean (SD)

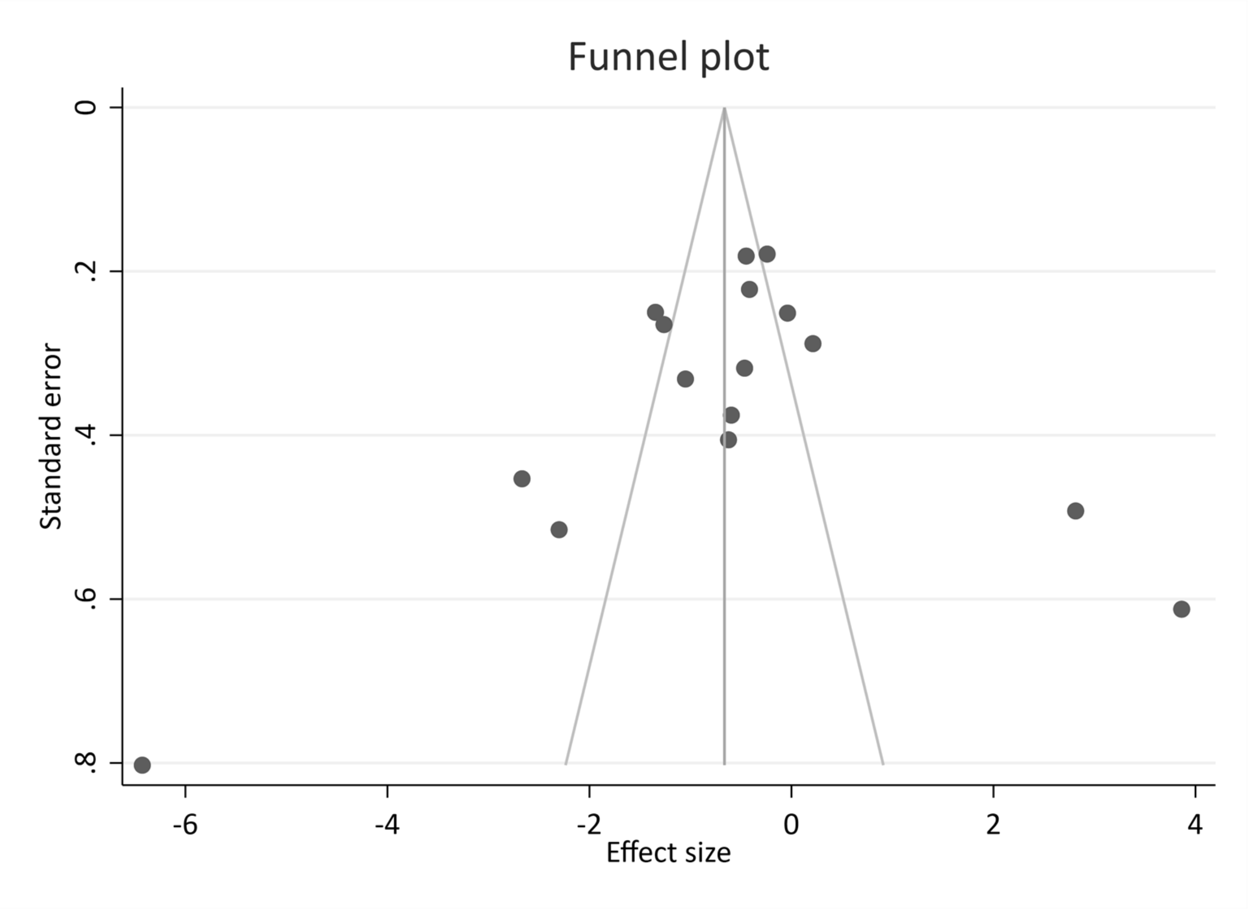

Figure 2: Funnel plot for active ghrelin (basal).

Total ghrelin (basal)

Table 2: Summary of contributing articles for total ghrelin (basal)

Author (year)	Study design	Obesity group n	% female	Age (years)	BMI (kg/m²)	Control group n	% female	Age (years)	BMI (kg/m²)
Acosta, 2015 [14]	Cross-sectional	201	72.0	37.8 (12.1)	35.4 (3.9)	105	61.0	37.3 (12.0)	27.6 (0.1)
Arafat, 2013 [26]	Parallel RCT	11	55.0	28.4 (8.6)	34.4 (5.6)	13	54.0	25.1 (2.2)	21.7 (2.2)
Batterham, 2003 [49]	Crossover	12	50.0	29.0 (8.3)	33.0 (3.1)	12	50.0	27.3 (1.4)	20.5 (0.3)
Bogdanov, 2019 [51]	Cross-sectional	15	80.0	38.7 (9.4)	41.2 (2.1)	15	87.0	37.0 (7.1)	21.8 (2.6)
Bowen, 2006 [52]	Crossover	47	0.0	56.8 (7.5)	30.1 (3.4)	25	0.0	50.5 (12.0)	23.3 (1.0)
Brownley, 2012 [53]	Crossover	20	50.0	34.9 (9.0)	34.7 (2.8)	20	50.0	27.9 (7.0)	22.9 (1.4)
Carlson, 2009 [54]	Cross-sectional	13	100.0	35.6 (9.7)	44.5 (7.1)	10	100.0	32.2 (8.6)	23.1 (1.3)
Cassar, 2015 [55]	Cross-sectional	18	100.0	35.0 (5.0)	31.0 (3.0)	22	100.0	28.0 (6.0)	22.0 (2.0)
Clamp, 2015 [84]	Cross-sectional	10	0.0	30.0 (6.0)	33.2 (3.0)	10	0.0	25.0 (5.0)	22.3 (1.6)
Cremonini, 2006 [85]	Parallel RCT	25	80.0	35.0 (8.0)	36.0 (4.0)	13	100.0	34.0 (12.0)	22.0 (2.0)
Daghestani, 2009 [57]	Cross-sectional	45	100.0	26.5 (6.4)	35.9 (6.2)	77	100.0	23.5 (4.5)	22.3 (3.3)
Druce, 2005 [86]	Parallel RCT	12	67.0	33.4 (6.9)	31.9 (3.5)	12	67.0	24.7 (4.6)	20.5 (0.6)
El Helou, 2019 [58]	Crossover	15	0.0	21.7 (3.5)	35.1 (3.9)	15	0.0	20.1 (1.5)	22.0 (1.9)
English, 2002 [28]	Cross-sectional	10	30.0	42.8 (7.0)	42.8 (6.4)	13	60.0	32.0 (8.3)	22.5 (1.9)
Erdmann, 2005 [59]	Cross-sectional	128	36.0	45.0 (13.6)	33.8 (5.7)	56	80.0	30.0 (10.5)	22.0 (2.2)
Espelund, 2005 [87]	Cross-sectional	16	56.0	39.7 (12.4)	29.5 (4.0)	17	47.0	33.7 (14.0)	23.4 (2.5)
Frecka, 2008 [88]	Cross-sectional	5	40.0	32.0 (9.6)	32.2 (1.6)	7	57.0	24.3 (4.2)	23.3 (1.6)
Guo, 2007 [60]	Cross-sectional	14	50.0	59.4 (7.3)	30.1 (1.9)	16	50.0	54.4 (4.5)	21.6 (1.7)
Haltia, 2010 [89]	Crossover	13	38.0	27.0 (6.0)	33.0 (4.5)	12	50.0	26.0 (5.0)	21.7 (1.3)
Karcz-Socha, 2011 [45]	Cross-sectional	96	50.0	51.5 (6.5)	35.3 (2.9)	46	52.0	51.2 (6.5)	23.4 (1.5)
Kheirouri, 2017 [62]	Cross-sectional	37	100.0	37.2 (7.5)	23.1 (0.8)	40	100.0	35.2 (7.9)	31.4 (0.7)
Kiessl, 2017 [63]	Crossover	43	100.0	37.2 (7.5)	31.5 (1.8)	42	100.0	35.2 (7.9)	21.7 (2.0)
Kocak, 2011 [64]	Cross-sectional	22	100.0	NR	34.1 (4.0)	19	100.0	NR	25.2 (1.7)
Kolodziejski, 2018 [43]	Cross-sectional	15	100.0	53.0 (8.5)	39.8 (1.0)	15	100.0	58.6 (10.3)	22.3 (0.5)
Korek, 2013 [32]	Cross-sectional	19	90.0	42.2 (3.3)	34.7 (4.9)	17	89.0	42.9 (5.3)	21.1 (1.9)
Korner, 2005 [90]	Cross-sectional	12	100.0	NR	34.1 (1.8)	8	100.0	NR	21.6 (0.7)
Lambert, 2011 [91]	Crossover	11	NR	32.5 (6.5)	29.3 (0.6)	11	NR	28.8 (4.8)	21.3 (0.6)
Leonetti, 2003 [65]	Cross-sectional	8	70.0	20.8 (0.6)	35.9 (2.5)	10	50.0	21.5 (0.5)	23.0 (3.6)
Marzullo, 2006 [17]	Cross-sectional	10	50.0	32.4 (1.6)	43.0 (0.9)	6	50.0	31.7 (1.3)	21.8 (1.4)
Outeiriño-Blanco, 2011 [67]	Cross-sectional	23	100.0	39.8 (2.9)	38.8 (1.2)	13	100.0	34.4 (3.6)	22.3 (0.7)
Ozkan, 2009 [68]	Parallel RCT	21	71.0	37.4 (12.4)	24.8 (3.1)	10	60.0	36.1 (9.8)	37.0 (3.6)
Papandreou, 2017 [69]	Cross-sectional	7	NR	21.0 (2.1)	31.3 (2.1)	13	NR	21.0 (0.8)	20.6 (3.0)
Pavlatos, 2005 [70]	Parallel RCT	9	100.0	39.5 (14.5)	37.2 (8.4)	9	100.0	38.7 (14.1)	23.0 (2.1)

BMI: Body mass index; NR: not reported; Values for age and BMI are presented as mean (SD)

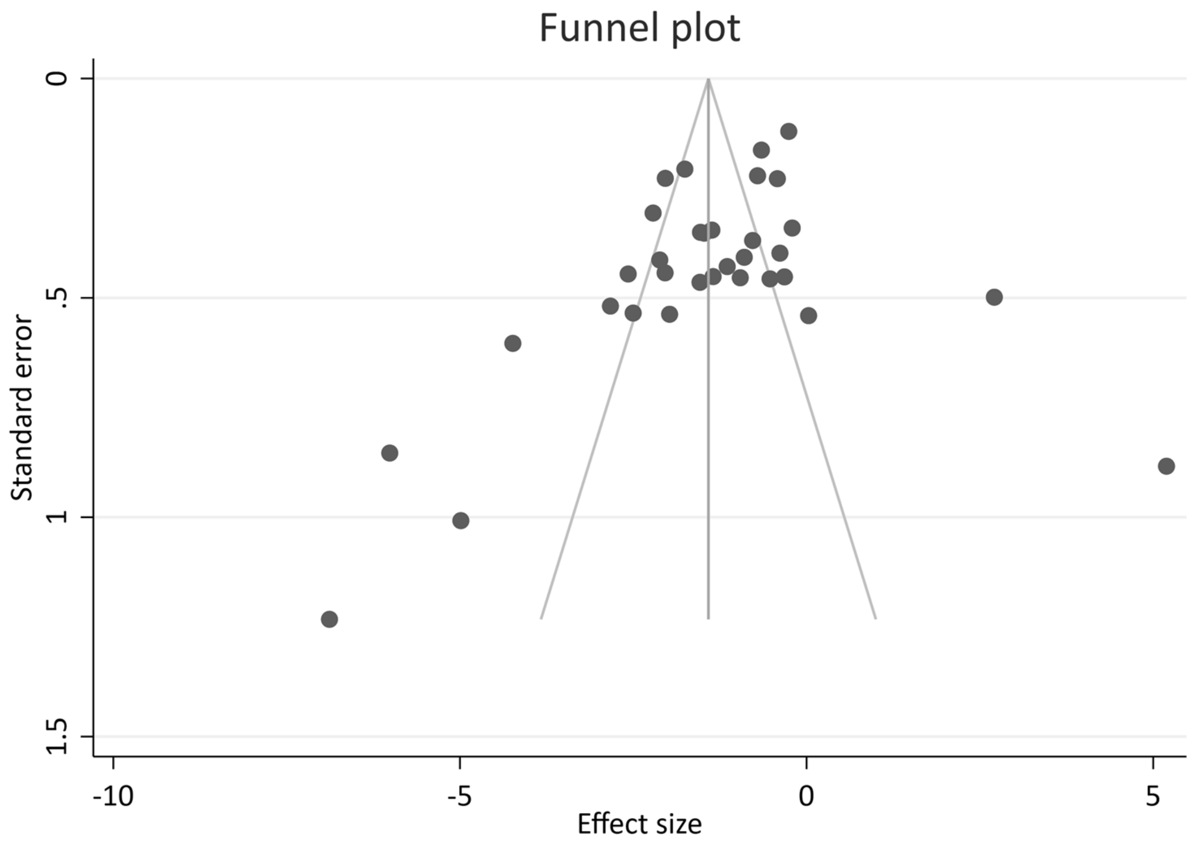

Figure 3: Funnel plot for total ghrelin (basal).

Active GLP-1 (basal)

Table 3: Summary of contributing articles for active GLP-1 (basal)

		Obesity group				Control group			
**Author (year)**	**Study design**	**n**	**% female**	**Age (years)**	**BMI (kg/m²)**	**n**	**% female**	**Age (years)**	**BMI (kg/m²)**
Adam, 2005 [94]	Crossover	28	68.0	44.4 (9.8)	30.4 (2.7)	30	50.0	31.6 (12.8)	22.9 (1.5)
Bowen, 2006 [52]	Crossover	47	0.0	56.8 (7.5)	30.1 (3.4)	25	0.0	50.5 (12.0)	23.3 (1.0)
Calanna, 2013 [95]	Cross-sectional	43	67.0	42.8 (13.1)	34.6 (3.9)	24	83.0	38.3 (9.8)	22.1 (2.0)
Carrol, 2007 [40]	Cross-sectional	20	55.0	47.2 (11.0)	36.8 (4.8)	19	47.0	39.2 (10.3)	22.6 (1.2)
DeBenedictis, 2020 [16]	Cross-sectional	34	50.0	38.9 (2.0)	34.0 (0.4)	33	49.0	45.0 (1.5)	24.8 (0.4)
Mersebach, 2003 [98]	Cross-sectional	16	75.0	39.3 (3.5)	36.1 (2.4)	10	70.0	37.9 (5.9)	21.5 (1.3)
Smith, 2021 [103]	Crossover	12	0.0	34.8 (7.4)	33.7 (2.4)	12	0.0	35.8 (10.6)	23.7 (1.8)

BMI: Body mass index; NR: not reported; Values for age and BMI are presented as mean (SD)

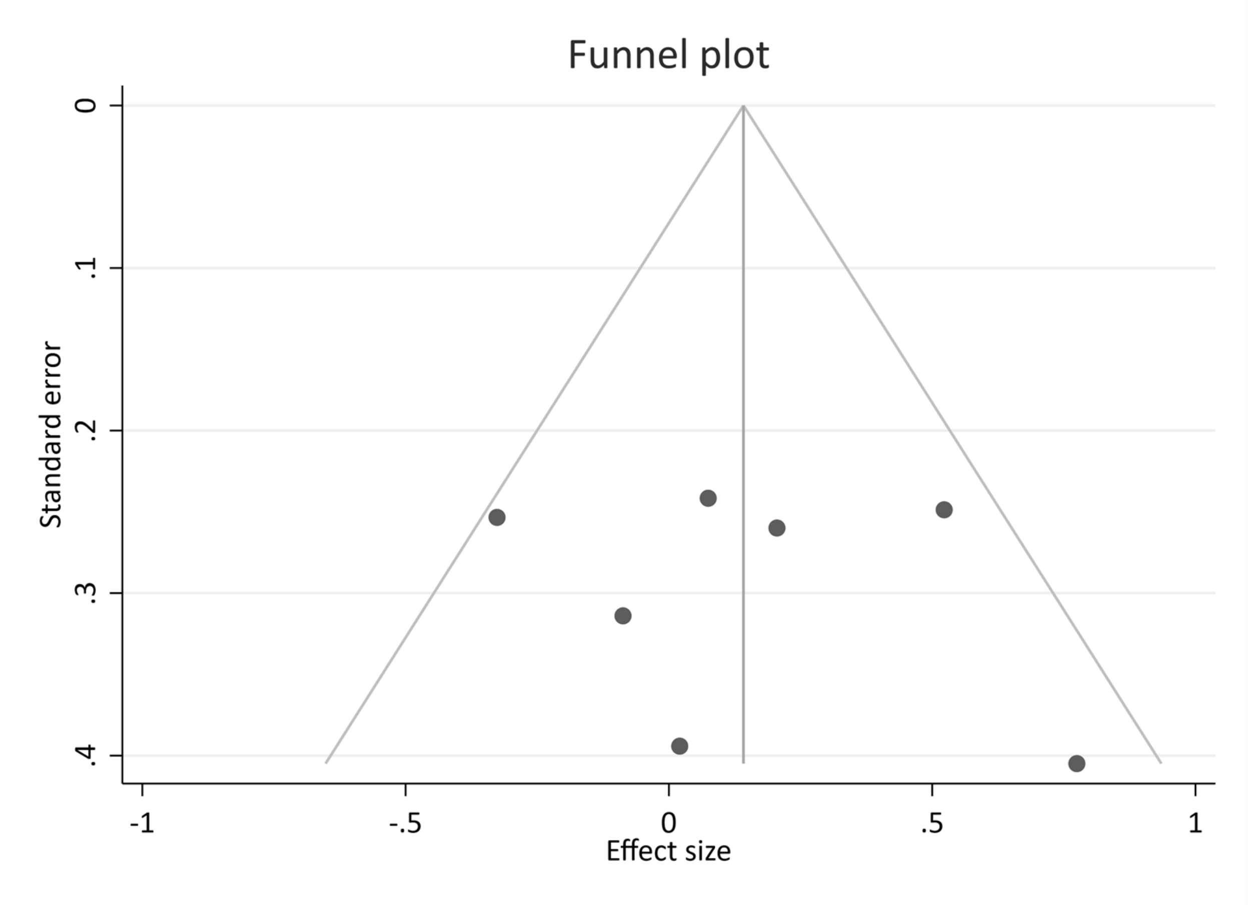

Figure 4: Funnel plot for active GLP-1 (basal).

Total GLP-1 (basal)

Table 4: Summary of contributing articles for total GLP-1 (basal)

Author (year)	Study design	Obesity group n	% female	Age (years)	BMI (kg/m²)	Control group n	% female	Age (years)	BMI (kg/m²)
Carr, 2010 [105]	Crossover	13	0.0	25.6 (4.0)	33.8 (0.6)	12	0.0	22.0 (1.8)	22.3 (0.3)
Cassar, 2015 [55]	Cross-sectional	18	100.0	35.0 (5.0)	31.0 (3.0)	22	100.0	28.0 (6.0)	22.0 (2.0)
Chia, 2017 [106]	Cross-sectional	20	50.0	68.5 (13.0)	35.6 (4.0)	20	50.0	68.5 (13.0)	23.6 (1.3)
Dirksen, 2019 [104]	Crossover	10	0.0	42.1 (9.8)	57.6 (17.2)	10	0.0	43.1 (8.9)	24.1 (3.8)
Douglas, 2017 [42]	Crossover	25	50.0	45.0 (12.4)	29.2 (2.9)	22	44.0	37.5 (15.2)	22.4 (1.5)
El Helou, 2019 [58]	Crossover	15	0.0	21.7 (3.5)	35.1 (3.9)	15	0.0	20.1 (1.5)	22.0 (1.9)
Elahi, 2016 [96]	Cross-sectional	12	50.0	42.0 (6.9)	37.2 (5.2)	12	50.0	29.0 (6.4)	22.3 (1.4)
Greenfield, 2009 [108]	Crossover	8	14.0	39.0 (9.8)	34.5 (4.4)	8	33.0	30.0 (5.8)	21.9 (2.2)
Haltia, 2010 [89]	Crossover	13	38.0	27.0 (6.0)	33.0 (4.5)	12	50.0	26.0 (5.0)	21.7 (1.3)
Heni, 2015 [109]	Cross-sectional	12	50.0	25.0 (6.9)	30.5 (6.2)	12	50.0	23.0 (6.9)	21.2 (3.8)
Nguyen, 2018 [110]	Cross-sectional	22	50.0	50.2 (2.5)	48.6 (1.8)	10	59.0	38.6 (8.4)	23.9 (0.7)
Smith, 2021 [103]	Crossover	12	0.0	34.8 (7.4)	33.7 (2.4)	12	0.0	35.8 (10.6)	23.7 (1.8)
Verdich, 2001 [113]	.	19	0.0	35.0 (10.9)	38.1 (3.1)	12	0.0	24.2 (9.6)	23.1 (1.4)

BMI: Body mass index; NR: not reported; Values for age and BMI are presented as mean (SD)

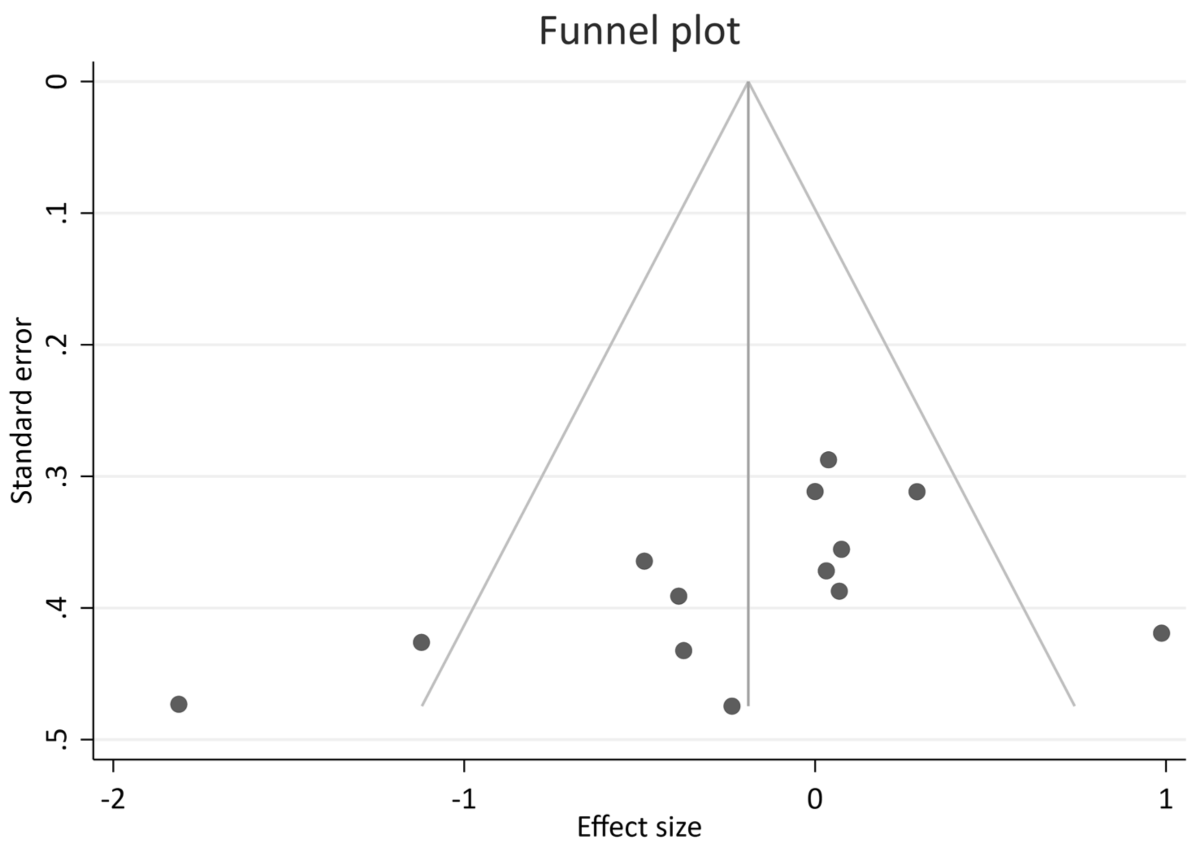

Figure 5: Funnel plot for total GLP-1 (basal).

Active PYY (basal)

Table 5: Summary of contributing articles for active PYY (basal)

		Obesity group				Control group			
**Author (year)**	**Study design**	**n**	**% female**	**Age (years)**	**BMI (kg/m²)**	**n**	**% female**	**Age (years)**	**BMI (kg/m²)**
Batterham, 2003 [49]	Crossover	12	50.0	29.0 (8.3)	33.0 (3.1)	12	50.0	27.3 (1.4)	20.5 (0.3)
Damgaard, 2013 [107]	Crossover	13	0.0	32.3 (7.7)	30.6 (3.5)	8	0.0	27.1 (6.1)	22.1 (2.3)
Dirksen, 2019 [104]	Crossover	10	0.0	42.1 (9.8)	57.6 (17.2)	10	0.0	43.1 (8.9)	24.1 (3.8)
Haltia, 2010 [89]	Crossover	13	38.0	27.0 (6.0)	33.0 (4.5)	12	50.0	26.0 (5.0)	21.7 (1.3)
Karcz-Socha, 2011 [45]	Cross-sectional	96	50.0	51.5 (6.5)	35.3 (2.9)	46	52.0	51.2 (6.5)	23.4 (1.5)
Korner, 2005 [90]	Cross-sectional	12	100.0	NR	34.1 (1.8)	8	100.0	NR	21.6 (0.7)
Outeiriño-Blanco, 2011 [67]	Cross-sectional	23	100.0	39.8 (2.9)	38.8 (1.2)	13	100.0	34.4 (3.6)	22.3 (0.7)
Pfluger, 2007 [117]	Crossover	15	100.0	52.0 (2.0)	31.1 (0.5)	17	100.0	51.6 (1.9)	22.0 (0.5)

BMI: Body mass index; NR: not reported; Values for age and BMI are presented as mean (SD)

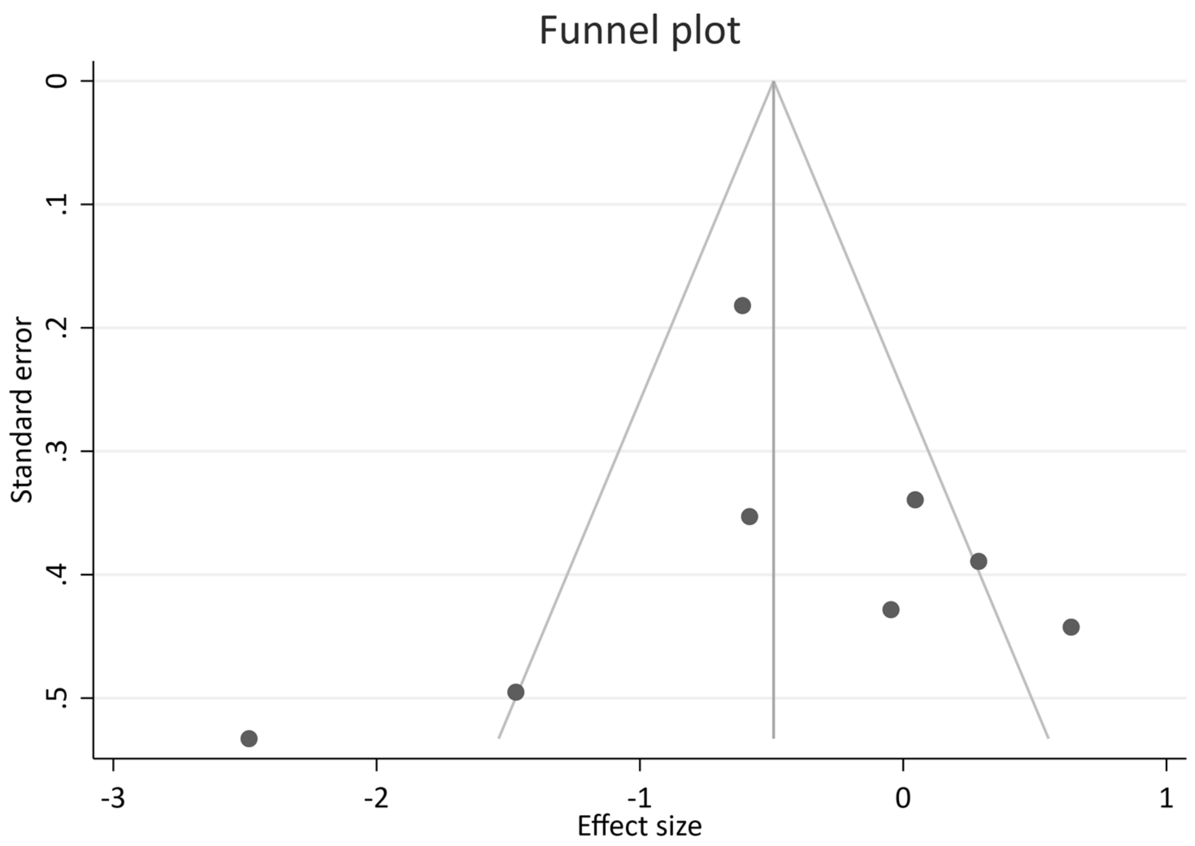

Figure 6: Funnel plot for active PYY (basal).

Total PYY (basal)

Table 6: Summary of contributing articles for total PYY (basal)

Author (year)	Study design	Obesity group				Control group			
		n	% female	Age (years)	BMI (kg/m²)	n	% female	Age (years)	BMI (kg/m²)
Brownley, 2010 [120]	Crossover	20	50.0	34.9 (9.0)	34.7 (2.8)	20	50.0	27.9 (7.0)	22.9 (1.4)
Cahill, 2011 [124]	Cross-sectional	28	0.0	23.2 (2.6)	29.1 (4.9)	41	0.0	23.1 (3.5)	23.1 (2.3)
Cahill, 2014 [123]	Cross-sectional	779	74.0	43.8 (12.1)	30.6 (4.8)	1315	76.0	42.1 (13.2)	24.4 (3.1)
Clamp, 2015 [84]	Cross-sectional	10	0.0	30.0 (6.0)	33.2 (3.0)	10	0.0	25.0 (5.0)	22.3 (1.6)
DeBenedictis, 2020 [16]	Cross-sectional	34	50.0	38.9 (2.0)	34.0 (0.4)	33	49.0	45.0 (1.5)	24.8 (0.4)
Douglas, 2017 [42]	Crossover	25	50.0	45.0 (12.4)	29.2 (2.9)	22	44.0	37.5 (15.2)	22.4 (1.5)
Pfluger, 2007 [117]	Crossover	79	73.0	47.6 (2.9)	35.1 (4.8)	66	70.0	41.5 (2.2)	22.1 (0.2)

BMI: Body mass index; NR: not reported; Values for age and BMI are presented as mean (SD)

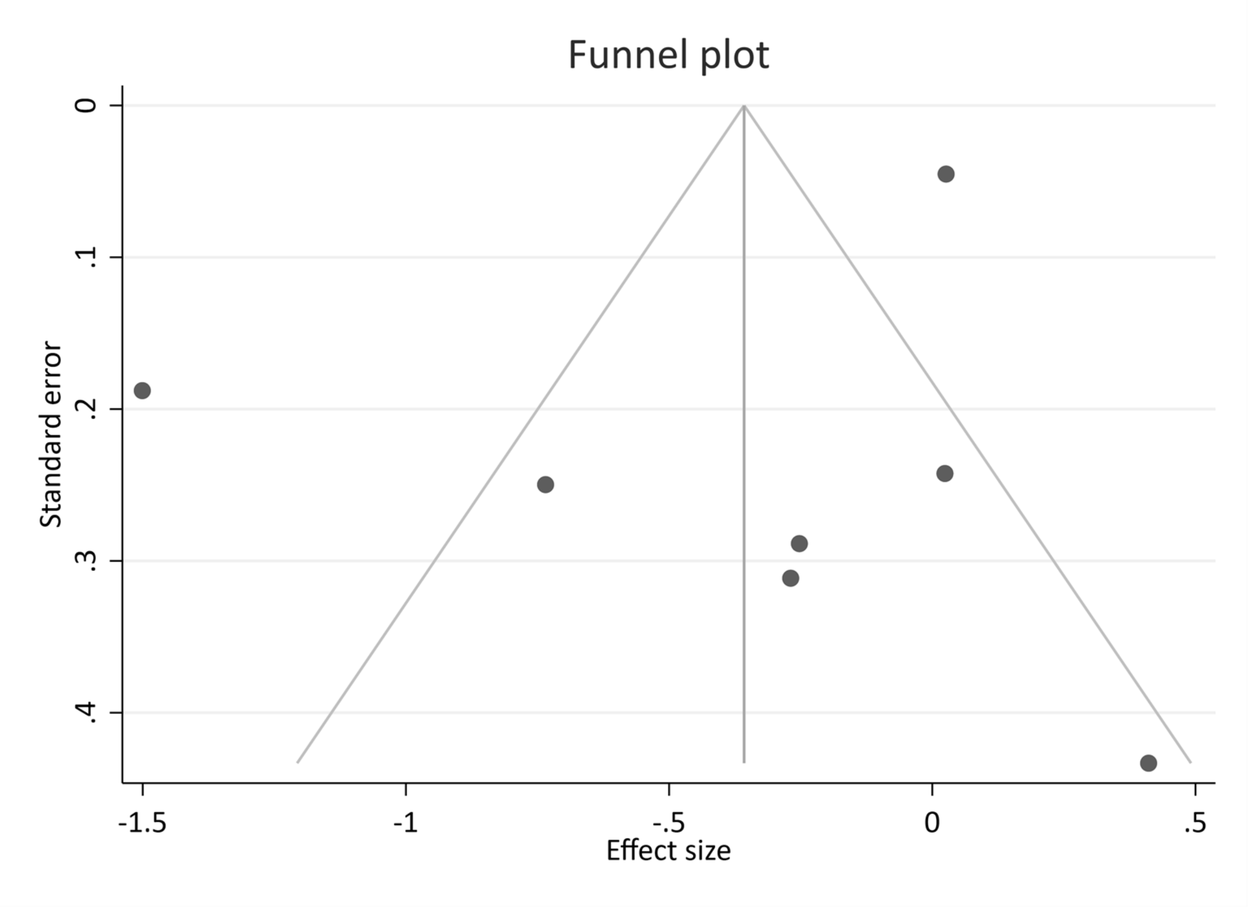

Figure 7: Funnel plot for total PYY (basal).

CKK (basal)

Table 7: Summary of contributing articles for CKK (basal)

Author (year)	Study design	Obesity group				Control group			
		n	% female	Age (years)	BMI (kg/m²)	n	% female	Age (years)	BMI (kg/m²)
Bowen, 2006 [52]	Crossover	47	0.0	56.8 (7.5)	30.1 (3.4)	25	0.0	50.5 (12.0)	23.3 (1.0)
DeBenedictis, 2020 [16]	Cross-sectional	34	50.0	38.9 (2.0)	34.0 (0.4)	33	49.0	45.0 (1.5)	24.8 (0.4)
Dirksen, 2019 [104]	Crossover	10	0.0	42.1 (9.8)	57.6 (17.2)	10	0.0	43.1 (8.9)	24.1 (3.8)
French, 1993 [18]	Crossover	8	63.0	NR	NR	7	57.0	NR	NR
Lieverse, 1993 [126]	Parallel RCT	7	100.0	40.2 (10.6)	40.7 (6.7)	7	100.0	41.2 (11.6)	22.3 (2.1)
Lieverse, 1998 [125]	Parallel RCT	7	100.0	NR	39.0 (2.0)	7	100.0	NR	22.0 (0.3)
Milewicz, 2000 [127]	Cross-sectional	25	NR	NR	34.6 (1.4)	16	NR	NR	21.2 (0.4)

BMI: Body mass index; NR: not reported; Values for age and BMI are presented as mean (SD)

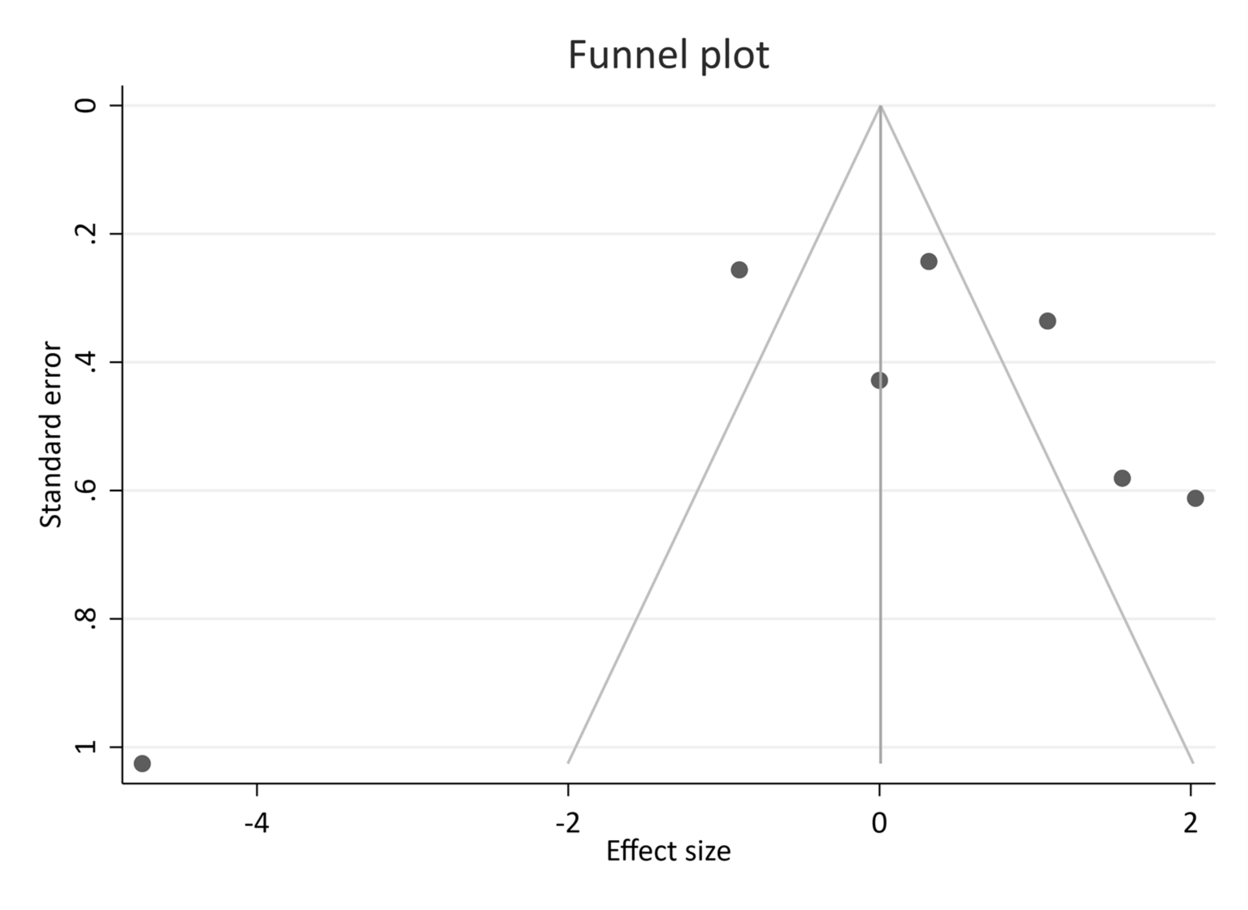

Figure 8: Funnel plot for CKK (basal)

Postprandial hormone concentrations

Total ghrelin (AUC)

Table 8: Summary of contributing articles for total ghrelin (AUC)

		Obesity group				Control group			
Author (year)	Study design	n	% female	Age (years)	BMI (kg/m²)	n	% female	Age (years)	BMI (kg/m²)
Brownley, 2012 [53]	Crossover	20	50.0	34.9 (9.0)	34.7 (2.8)	20	50.0	27.9 (7.0)	22.9 (1.4)
Frecka, 2008 [88]	Cross-sectional	5	40.0	32.0 (9.6)	32.2 (1.6)	7	57.0	24.3 (4.2)	23.3 (1.6)
Marzullo, 2006 [17]	Cross-sectional	10	50.0	32.4 (1.6)	43.0 (0.9)	6	50.0	31.7 (1.3)	21.8 (1.4)
Meyer-Gerspach, 2014 [15]	Cross-sectional	20	NR	29.8 (1.9)	39.3 (1.9)	20	NR	24.1 (0.6)	21.8 (0.4)
Outeiriño-Blanco, 2011 [67]	Cross-sectional	23	100.0	39.8 (2.9)	38.8 (1.2)	13	100.0	34.4 (3.6)	22.3 (0.7)

BMI: Body mass index; NR: not reported; Values for age and BMI are presented as mean (SD)

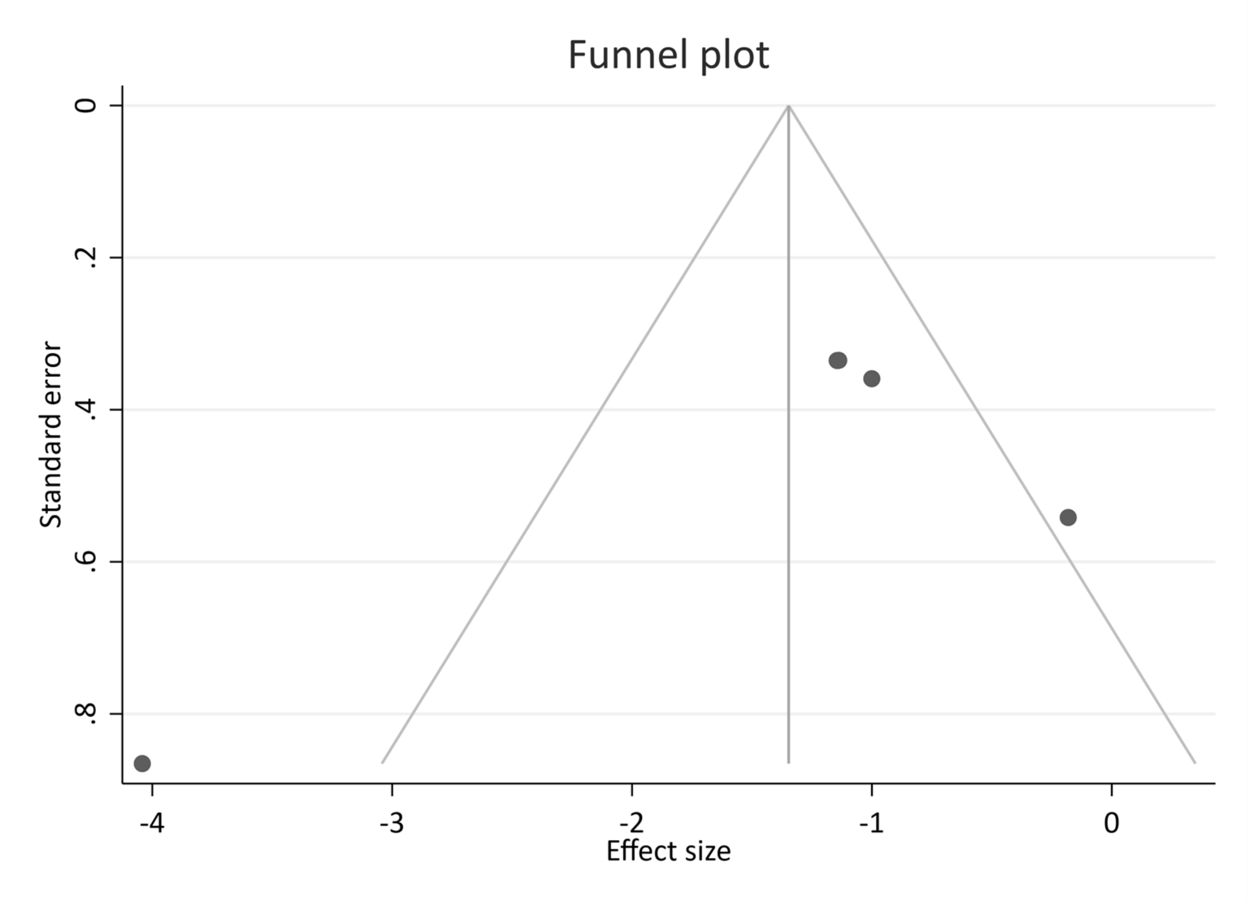

Figure 9: Funnel plot for total ghrelin (AUC).

Active GLP-1 (AUC)

Table 9: Summary of contributing articles for active GLP-1 (AUC)

Author (year)	Study design	Obesity group				Control group			
		n	% female	Age (years)	BMI (kg/m²)	n	% female	Age (years)	BMI (kg/m²)
Adam, 2005 [94]	Crossover	28	68.0	44.4 (9.8)	30.4 (2.7)	30	50.0	31.6 (12.8)	22.9 (1.5)
DeBenedictis, 2020 [16]	Cross-sectional	34	50.0	38.9 (2.0)	34.0 (0.4)	33	49.0	45.0 (1.5)	24.8 (0.4)
Heden, 2013 [97]	Crossover	13	54.0	25.4 (3.6)	34.6 (3.6)	13	46.0	26.0 (7.2)	23.0 (1.8)
Meyer-Gerspach, 2014 [15]	Cross-sectional	20	NR	29.8 (1.9)	39.3 (1.9)	20	NR	24.1 (0.6)	21.8 (0.4)

BMI: Body mass index; NR: not reported; Values for age and BMI are presented as mean (SD)

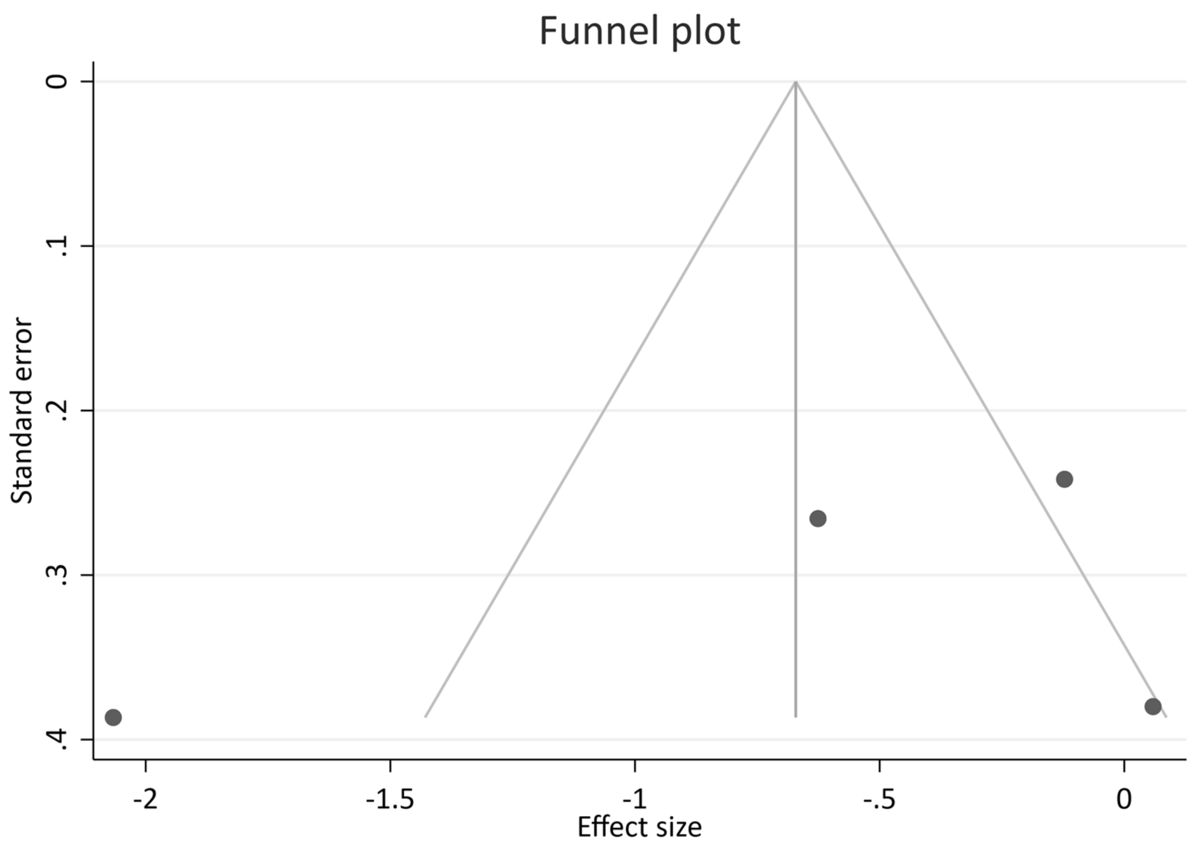

Figure 10: Funnel plot for active GLP-1 (AUC).

Total PYY (AUC)

Table 10: Summary of contributing articles for total PYY (AUC)

		Obesity group				Control group			
**Author (year)**	**Study design**	**n**	**% female**	**Age (years)**	**BMI (kg/m²)**	**n**	**% female**	**Age (years)**	**BMI (kg/m²)**
Brownley, 2010 [120]	Crossover	20	50.0	34.9 (9.0)	34.7 (2.8)	20	50.0	27.9 (7.0)	22.9 (1.4)
DeBenedictis, 2020 [16]	Cross-sectional	34	50.0	38.9 (2.0)	34.0 (0.4)	33	49.0	45.0 (1.5)	24.8 (0.4)
Meyer-Gerspach, 2014 [15]	Cross-sectional	20	NR	29.8 (1.9)	39.3 (1.9)	20	NR	24.1 (0.6)	21.8 (0.4)

BMI: Body mass index; NR: not reported; Values for age and BMI are presented as mean (SD)

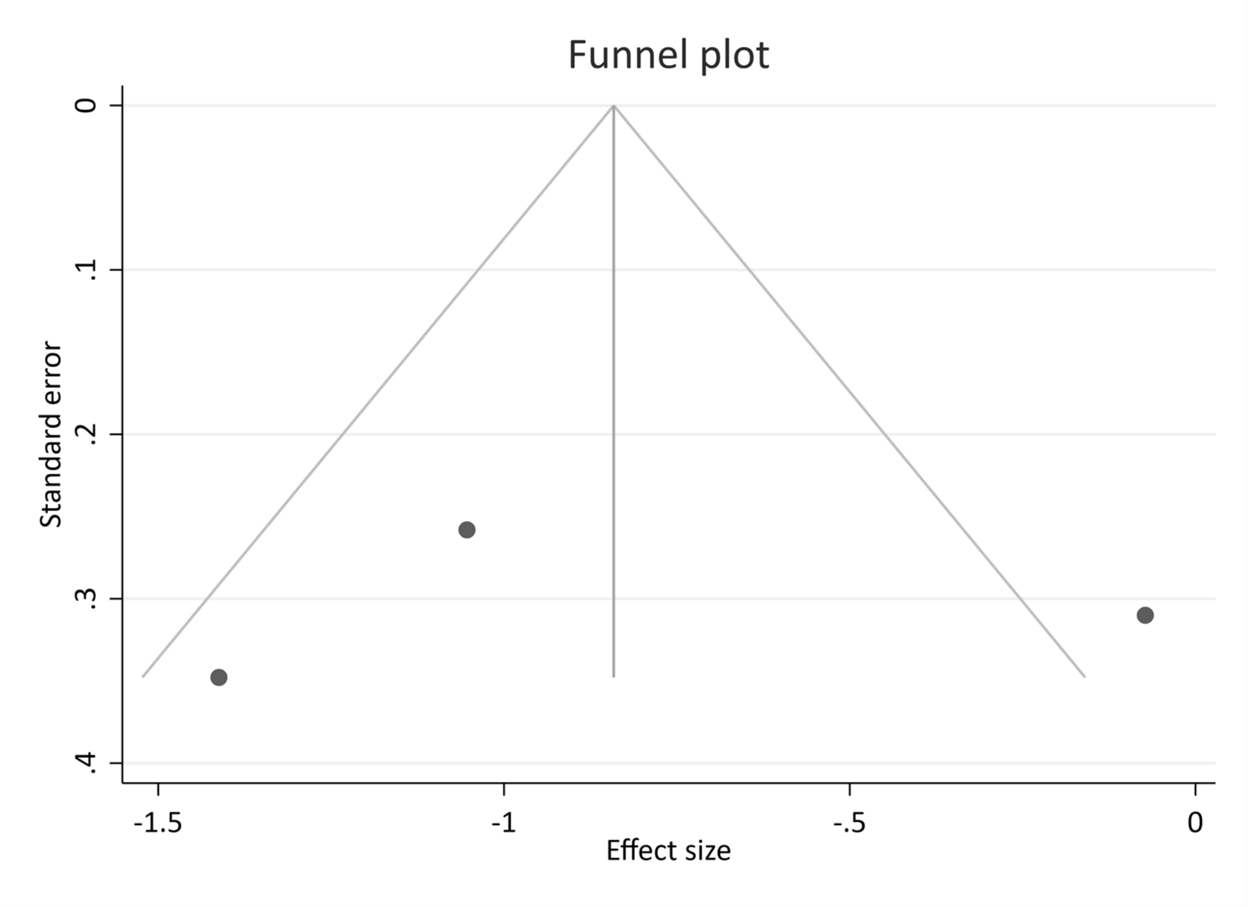

Figure 11: Funnel plot for total PYY (AUC).

Fasting appetite feelings

Hunger (fasting)

Table 11: Summary of contributing articles for hunger (fasting)

		Obesity group				Control group			
**Author (year)**	**Study design**	**n**	**% female**	**Age (years)**	**BMI (kg/m²)**	**n**	**% female**	**Age (years)**	**BMI (kg/m²)**
Andarini, 2017 [47]	Cross-sectional	16	0.0	21.4 (1.9)	33.6 (4.8)	16	0.0	20.6 (1.1)	21.3 (1.1)
Barkeling, 1995 [132]	Cross-sectional	38	50.0	43.5 (12.0)	39.4 (6.5)	38	50.0	40.0 (10.9)	22.3 (1.9)
Damgaard, 2013 [107]	Crossover	13	0.0	32.3 (7.7)	30.6 (3.5)	8	0.0	27.1 (6.1)	22.1 (2.3)
DeBenedictis, 2020 [16]	Cross-sectional	34	50.0	38.9 (2.0)	34.0 (0.4)	33	49.0	45.0 (1.5)	24.8 (0.4)
Dirksen, 2019 [104]	Crossover	10	0.0	42.1 (9.8)	57.6 (17.2)	10	0.0	43.1 (8.9)	24.1 (3.8)
El Helou, 2019 [58]	Crossover	15	0.0	21.7 (3.5)	35.1 (3.9)	15	0.0	20.1 (1.5)	22.0 (1.9)
Heden, 2013 [97]	Crossover	14	57.0	25.1 (5.0)	34.8 (4.4)	14	43.0	26.0 (6.0)	22.9 (1.7)
Iceta, 2019 [31]	Cross-sectional	55	100.0	38.0 (11.1)	41.5 (5.9)	29	100.0	37.0 (10.8)	21.5 (2.2)
Korner, 2005 [90]	Cross-sectional	12	100.0	NR	34.1 (1.8)	8	100.0	NR	21.6 (0.7)
Painchaud Guerard, 2016 [135]	Parallel RCT	51	55.0	44.2 (16.2)	33.1 (3.6)	302	53.0	36.7 (14.7)	24.2 (2.9)
Smith, 2021 [103]	Crossover	12	0.0	34.8 (7.4)	33.7 (2.4)	12	0.0	35.8 (10.6)	23.7 (1.8)

BMI: Body mass index; NR: not reported; Values for age and BMI are presented as mean (SD)

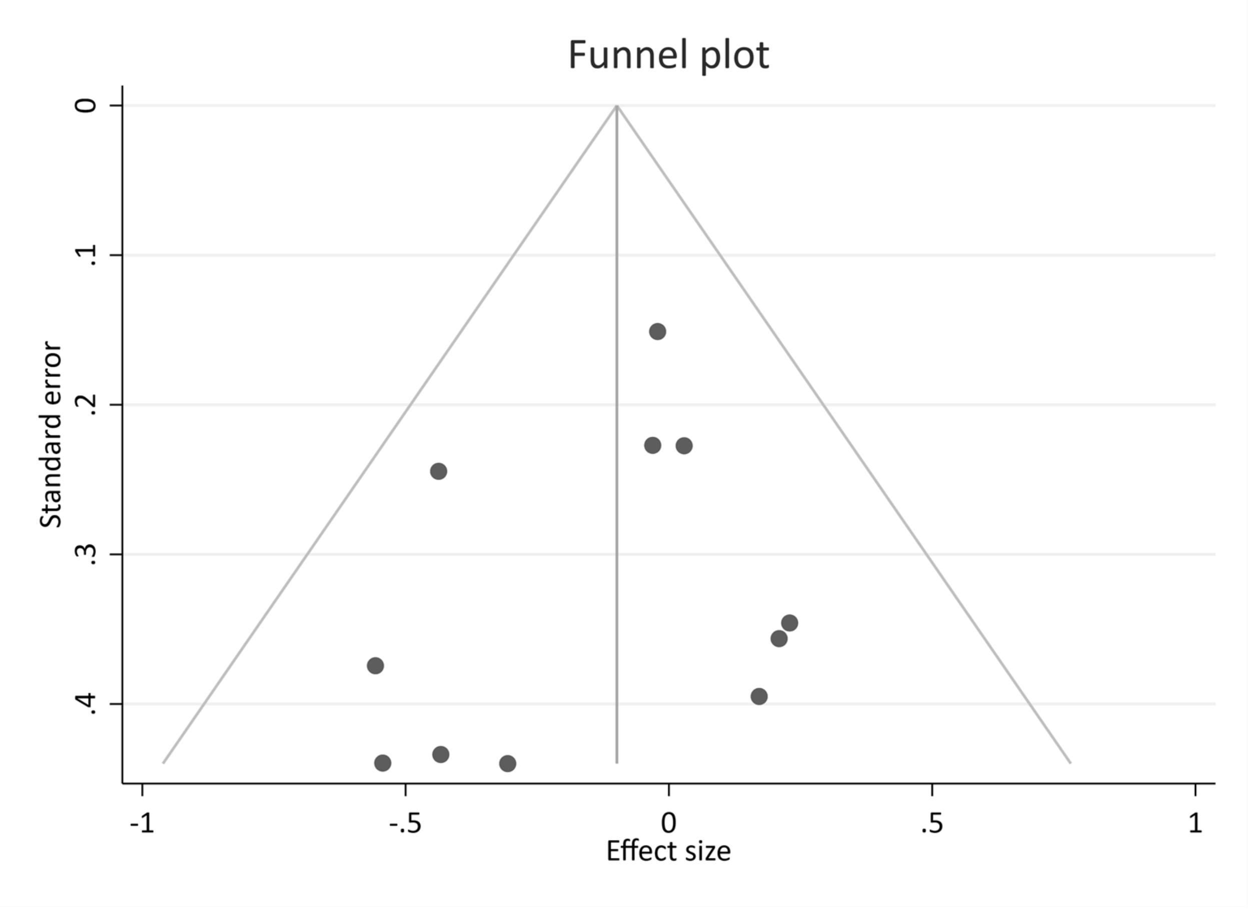

Figure 12: Funnel plot for hunger (fasting).

Fullness (fasting)

Table 12: Summary of contributing articles for fullness (fasting)

		Obesity group				Control group			
**Author (year)**	**Study design**	**n**	**% female**	**Age (years)**	**BMI (kg/m²)**	**n**	**% female**	**Age (years)**	**BMI (kg/m²)**
Barkeling, 1995 [132]	Cross-sectional	38	50.0	43.5 (12.0)	39.4 (6.5)	38	50.0	40.0 (10.9)	22.3 (1.9)
Damgaard, 2013 [107]	Crossover	13	0.0	32.3 (7.7)	30.6 (3.5)	8	0.0	27.1 (6.1)	22.1 (2.3)
DeBenedictis, 2020 [16]	Cross-sectional	34	50.0	38.9 (2.0)	34.0 (0.4)	33	49.0	45.0 (1.5)	24.8 (0.4)
Dirksen, 2019 [104]	Crossover	10	0.0	42.1 (9.8)	57.6 (17.2)	10	0.0	43.1 (8.9)	24.1 (3.8)
El Helou, 2019 [58]	Crossover	15	0.0	21.7 (3.5)	35.1 (3.9)	15	0.0	20.1 (1.5)	22.0 (1.9)
Heden, 2013 [97]	Crossover	14	57.0	25.1 (5.0)	34.8 (4.4)	14	43.0	26.0 (6.0)	22.9 (1.7)
Korner, 2005 [90]	Cross-sectional	12	100.0	NR	34.1 (1.8)	8	100.0	NR	21.6 (0.7)
Painchaud Guerard, 2016 [135]	Parallel RCT	51	55.0	44.2 (16.2)	33.1 (3.6)	302	53.0	36.7 (14.7)	24.2 (2.9)
Smith, 2021 [103]	Crossover	12	0.0	34.8 (7.4)	33.7 (2.4)	12	0.0	35.8 (10.6)	23.7 (1.8)

BMI: Body mass index; NR: not reported; Values for age and BMI are presented as mean (SD)

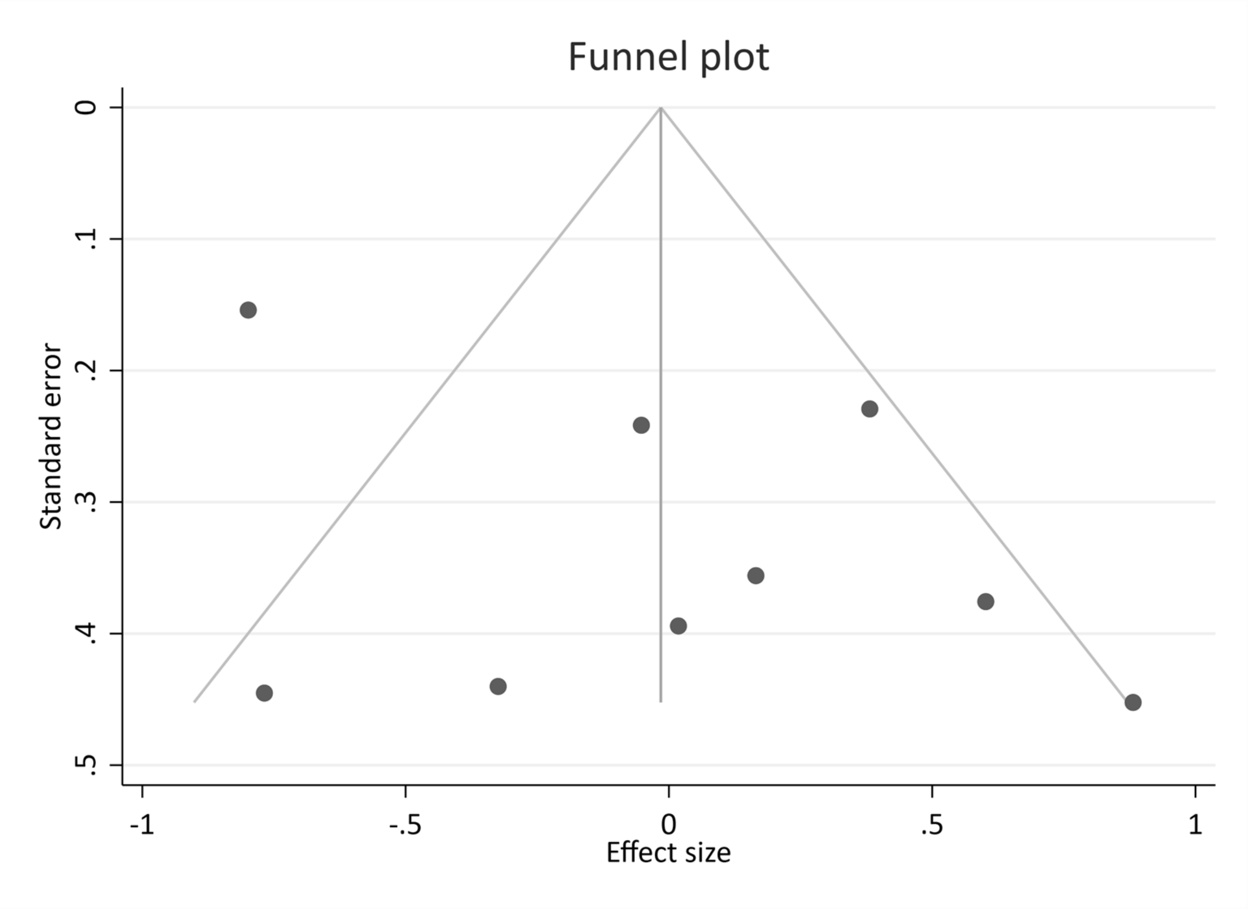

Figure 13: Funnel plot for fullness (fasting).

Desire to eat (DTE) (fasting)

Table 13: Summary of contributing articles for DTE (fasting)

Author (year)	Study design	Obesity group n	% female	Age (years)	BMI (kg/m²)	Control group n	% female	Age (years)	BMI (kg/m²)
Andarini, 2017 [47]	Cross-sectional	16	0.0	21.4 (1.9)	33.6 (4.8)	16	0.0	20.6 (1.1)	21.3 (1.1)
Barkeling, 1995 [132]	Cross-sectional	38	50.0	43.5 (12.0)	39.4 (6.5)	38	50.0	40.0 (10.9)	22.3 (1.9)
DeBenedictis, 2020 [16]	Cross-sectional	34	50.0	38.9 (2.0)	34.0 (0.4)	33	49.0	45.0 (1.5)	24.8 (0.4)
Painchaud Guerard, 2016 [135]	Parallel RCT	51	55.0	44.2 (16.2)	33.1 (3.6)	302	53.0	36.7 (14.7)	24.2 (2.9)

BMI: Body mass index; NR: not reported; Values for age and BMI are presented as mean (SD)

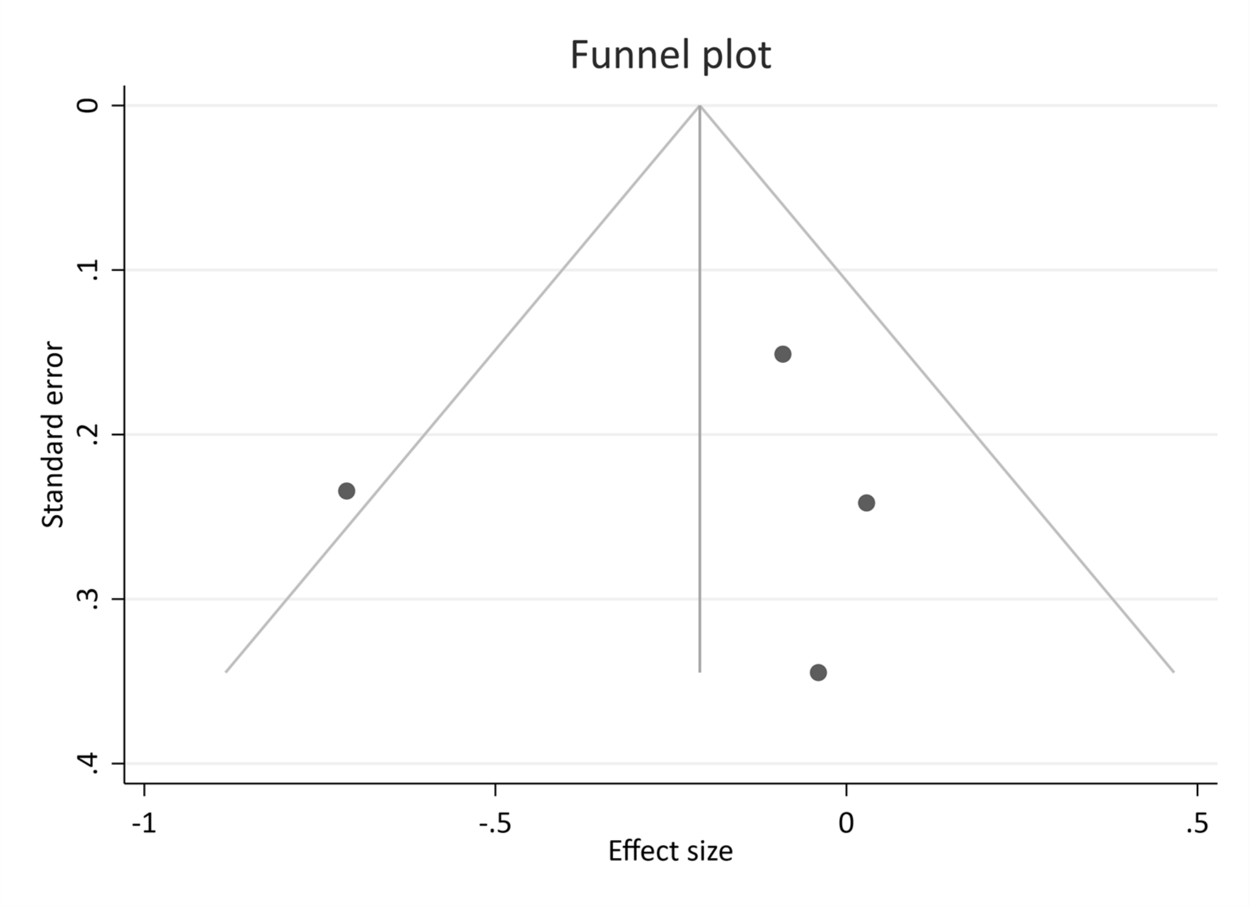

Figure 14: Funnel plot for DTE (fasting).

Prospective food consumption (PFC) (fasting)

Table 14: Summary of contributing articles for PFC (fasting)

		Obesity group				Control group			
**Author (year)**	**Study design**	**n**	**% female**	**Age (years)**	**BMI (kg/m²)**	**n**	**% female**	**Age (years)**	**BMI (kg/m²)**
Barkeling, 1995 [132]	Cross-sectional	38	50.0	43.5 (12.0)	39.4 (6.5)	38	50.0	40.0 (10.9)	22.3 (1.9)
DeBenedictis, 2020 [16]	Cross-sectional	34	50.0	38.9 (2.0)	34.0 (0.4)	33	49.0	45.0 (1.5)	24.8 (0.4)
El Helou, 2019 [58]	Crossover	15	0.0	21.7 (3.5)	35.1 (3.9)	15	0.0	20.1 (1.5)	22.0 (1.9)
Painchaud Guerard, 2016 [135]	Parallel RCT	51	55.0	44.2 (16.2)	33.1 (3.6)	302	53.0	36.7 (14.7)	24.2 (2.9)
Smith, 2021 [103]	Crossover	12	0.0	34.8 (7.4)	33.7 (2.4)	12	0.0	35.8 (10.6)	23.7 (1.8)

BMI: Body mass index; NR: not reported; Values for age and BMI are presented as mean (SD)

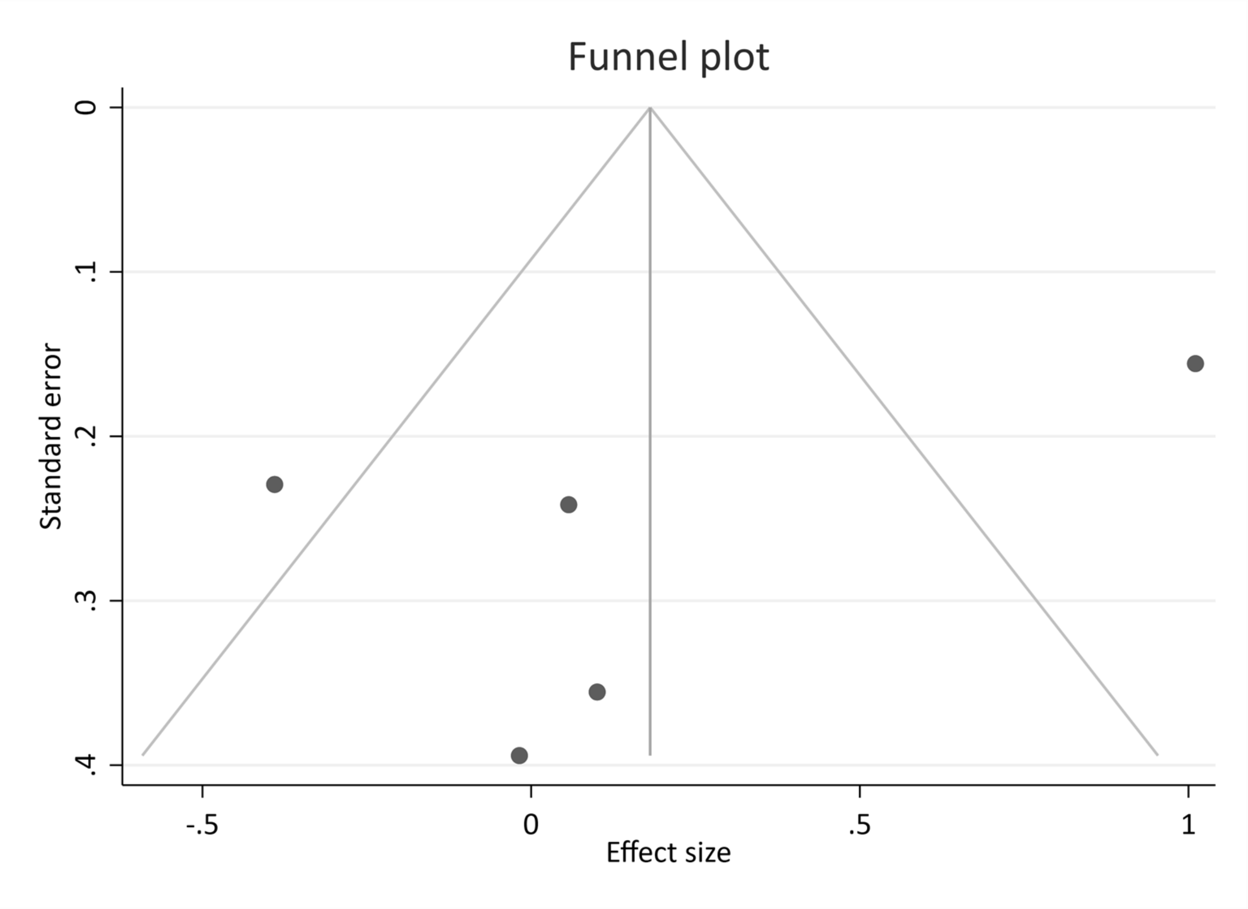

Figure 15: Funnel plot for PFC (fasting).

Postprandial appetite ratings

Hunger (AUC)

Table 15: Summary of contributing articles for hunger (AUC)

		Obesity group				Control group			
**Author (year)**	**Study design**	**n**	**% female**	**Age (years)**	**BMI (kg/m²)**	**n**	**% female**	**Age (years)**	**BMI (kg/m²)**
Andarini, 2017 [47]	Cross-sectional	16	0.0	21.4 (1.9)	33.6 (4.8)	16	0.0	20.6 (1.1)	21.3 (1.1)
DeBenedictis, 2020 [16]	Cross-sectional	34	50.0	38.9 (2.0)	34.0 (0.4)	33	49.0	45.0 (1.5)	24.8 (0.4)
Frecka, 2008 [88]	Cross-sectional	5	40.0	32.0 (9.6)	32.2 (1.6)	7	57.0	24.3 (4.2)	23.3 (1.6)
Painchaud Guerard, 2016 [135]	Parallel RCT	51	55.0	44.2 (16.2)	33.1 (3.6)	302	53.0	36.7 (14.7)	24.2 (2.9)
Smith, 2021 [103]	Crossover	12	0.0	34.8 (7.4)	33.7 (2.4)	12	0.0	35.8 (10.6)	23.7 (1.8)

BMI: Body mass index; NR: not reported; Values for age and BMI are presented as mean (SD)

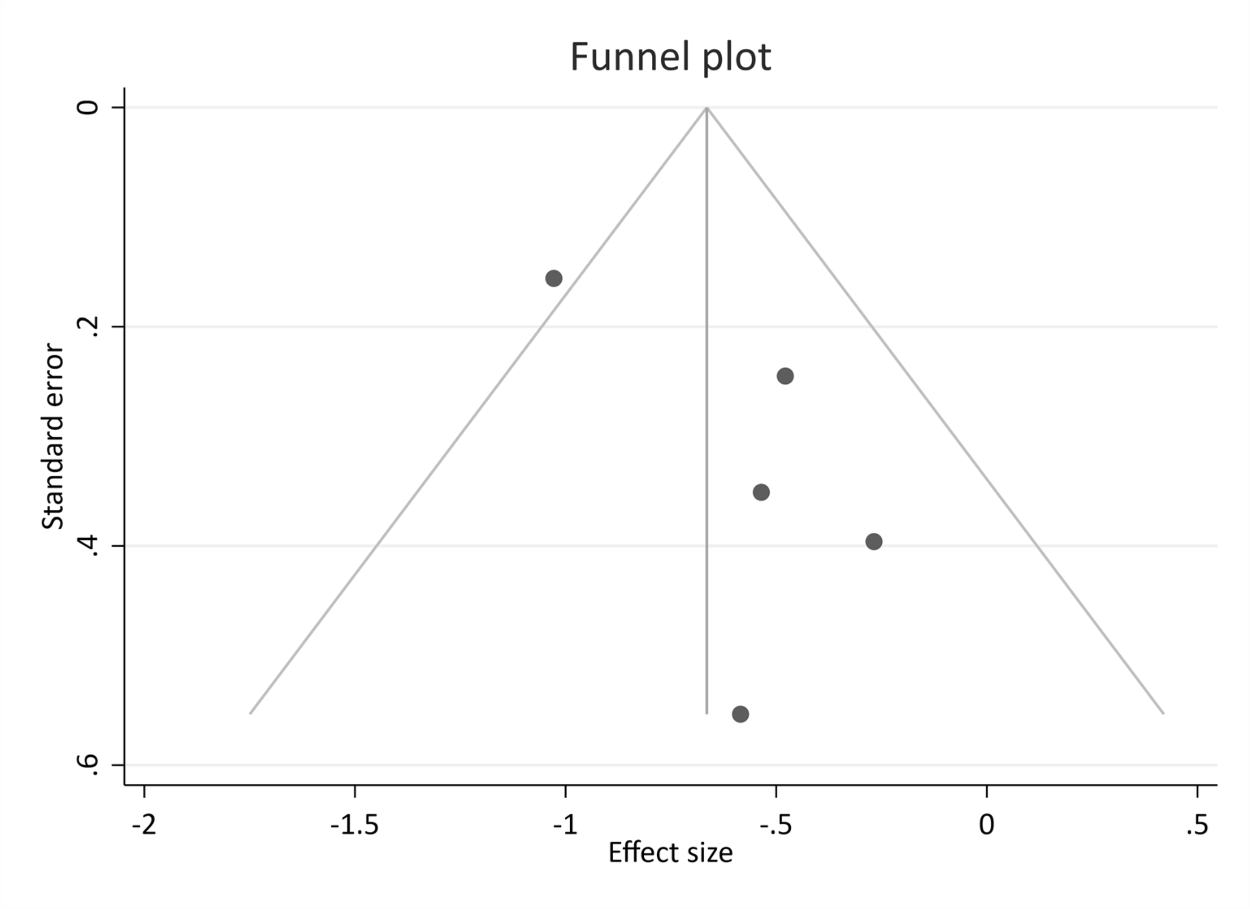

Figure 16: Funnel plot for hunger (AUC).

Fullness (AUC)

Table 16: Summary of contributing articles for fullness (AUC)

		Obesity group				Control group			
**Author (year)**	**Study design**	**n**	**% female**	**Age (years)**	**BMI (kg/m²)**	**n**	**% female**	**Age (years)**	**BMI (kg/m²)**
DeBenedictis, 2020 [16]	Cross-sectional	34	50.0	38.9 (2.0)	34.0 (0.4)	33	49.0	45.0 (1.5)	24.8 (0.4)
Frecka, 2008 [88]	Cross-sectional	5	40.0	32.0 (9.6)	32.2 (1.6)	7	57.0	24.3 (4.2)	23.3 (1.6)
Painchaud Guerard, 2016 [135]	Parallel RCT	51	55.0	44.2 (16.2)	33.1 (3.6)	302	53.0	36.7 (14.7)	24.2 (2.9)
Smith, 2021 [103]	Crossover	12	0.0	34.8 (7.4)	33.7 (2.4)	12	0.0	35.8 (10.6)	23.7 (1.8)

BMI: Body mass index; NR: not reported; Values for age and BMI are presented as mean (SD)

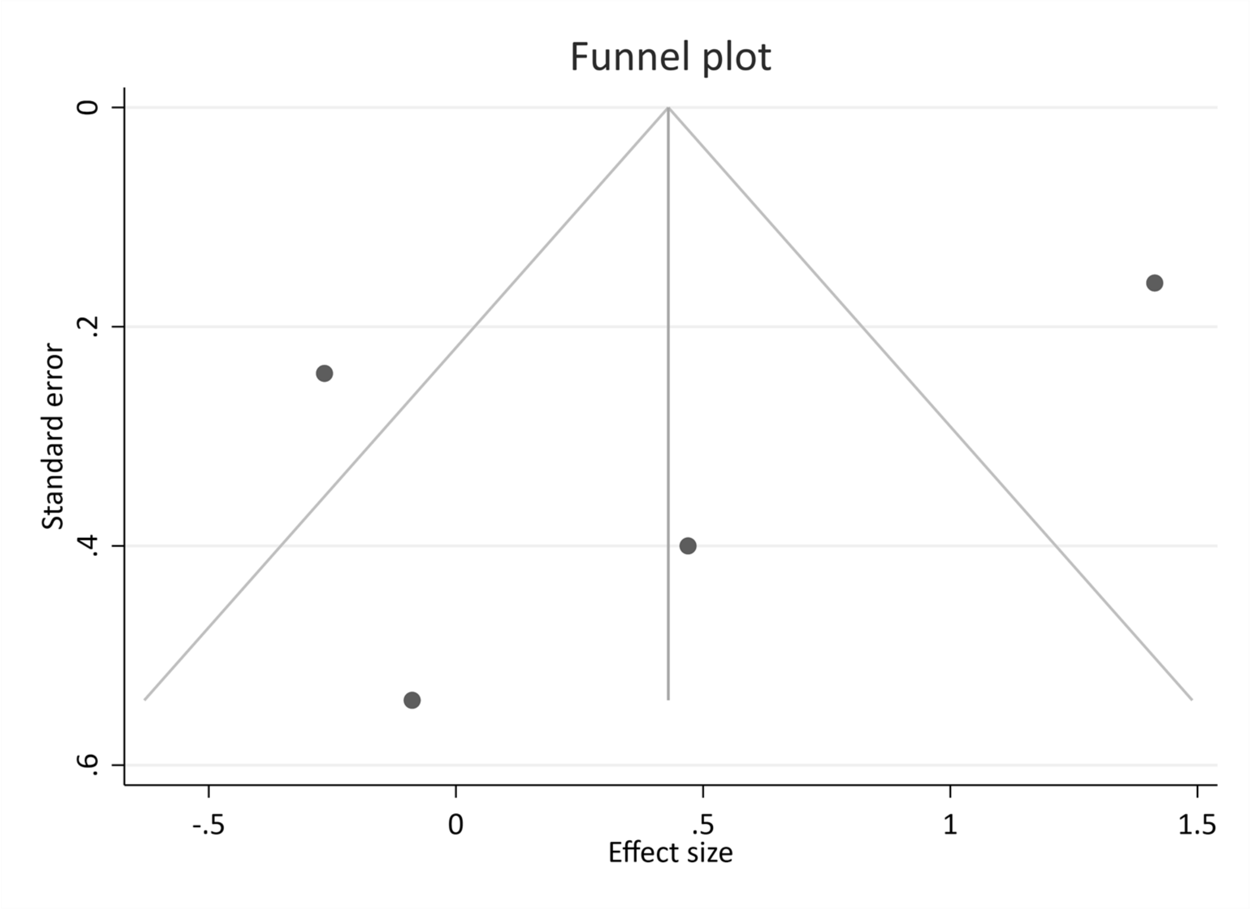

Figure 17: Funnel plot for fullness (AUC).

Desire to eat (DTE) (AUC)

Table 17: Summary of contributing articles for DTE (AUC)

		Obesity group				Control group			
**Author (year)**	**Study design**	**n**	**% female**	**Age (years)**	**BMI (kg/m^2)**	**n**	**% female**	**Age (years)**	**BMI (kg/m^2)**
Andarini, 2017 [47]	Cross-sectional	16	0.0	21.4 (1.9)	33.6 (4.8)	16	0.0	20.6 (1.1)	21.3 (1.1)
DeBenedictis, 2020 [16]	Cross-sectional	34	50.0	38.9 (2.0)	34.0 (0.4)	33	49.0	45.0 (1.5)	24.8 (0.4)
Frecka, 2008 [88]	Cross-sectional	5	40.0	32.0 (9.6)	32.2 (1.6)	7	57.0	24.3 (4.2)	23.3 (1.6)
Painchaud Guerard, 2016 [135]	Parallel RCT	51	55.0	44.2 (16.2)	33.1 (3.6)	302	53.0	36.7 (14.7)	24.2 (2.9)

BMI: Body mass index; NR: not reported; Values for age and BMI are presented as mean (SD)

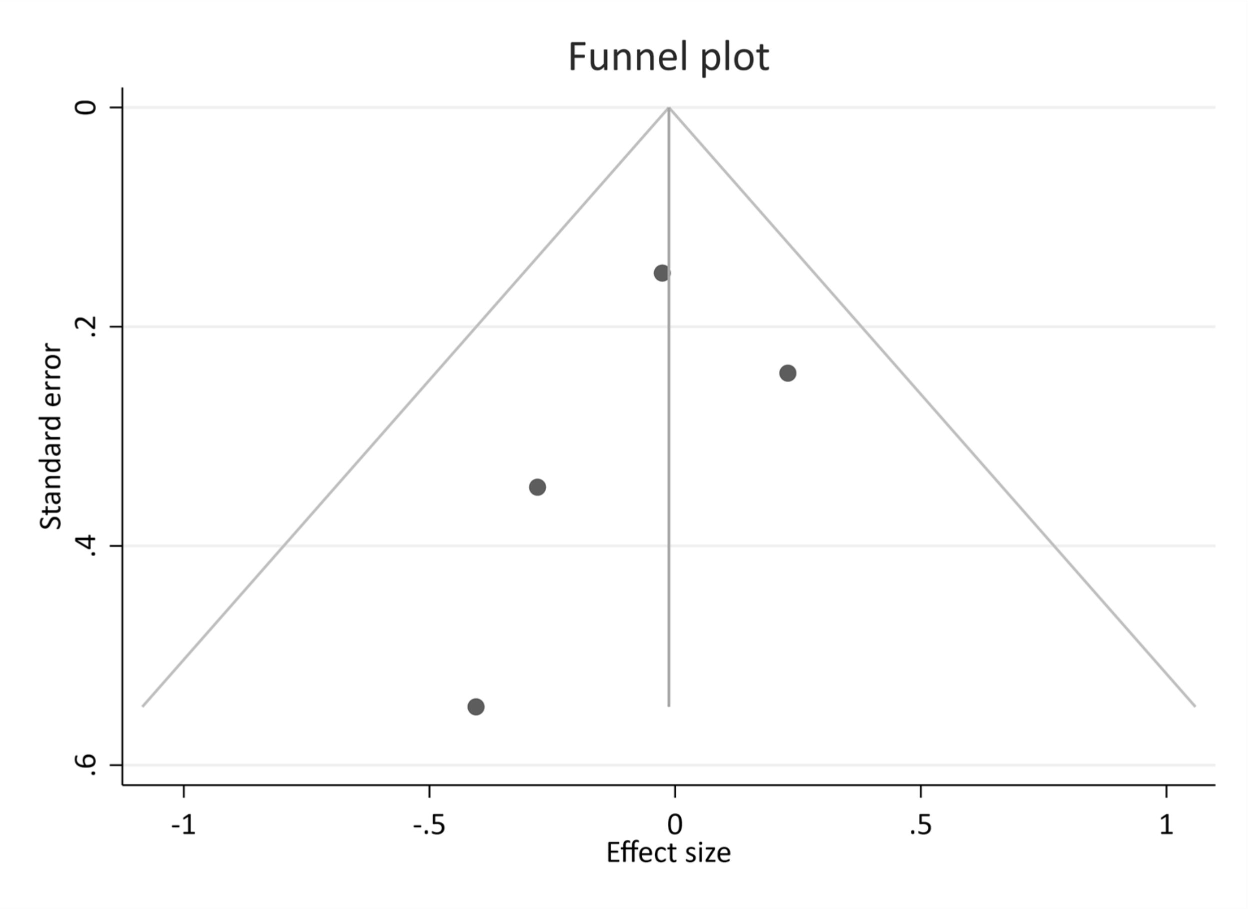

Figure 18: Funnel plot for DTE (AUC).

Prospective food consumption (PFC) (AUC)

Table 18: Summary of contributing articles for PFC (AUC)

		Obesity group				Control group			
**Author (year)**	**Study design**	**n**	**% female**	**Age (years)**	**BMI (kg/m^2)**	**n**	**% female**	**Age (years)**	**BMI (kg/m^2)**
DeBenedictis, 2020 [16]	Cross-sectional	34	50.0	38.9 (2.0)	34.0 (0.4)	33	49.0	45.0 (1.5)	24.8 (0.4)
Frecka, 2008 [88]	Cross-sectional	5	40.0	32.0 (9.6)	32.2 (1.6)	7	57.0	24.3 (4.2)	23.3 (1.6)
Painchaud Guerard, 2016 [135]	Parallel RCT	51	55.0	44.2 (16.2)	33.1 (3.6)	302	53.0	36.7 (14.7)	24.2 (2.9)
Smith, 2021 [103]	Crossover	12	0.0	34.8 (7.4)	33.7 (2.4)	12	0.0	35.8 (10.6)	23.7 (1.8)

BMI: Body mass index; NR: not reported; Values for age and BMI are presented as mean (SD)

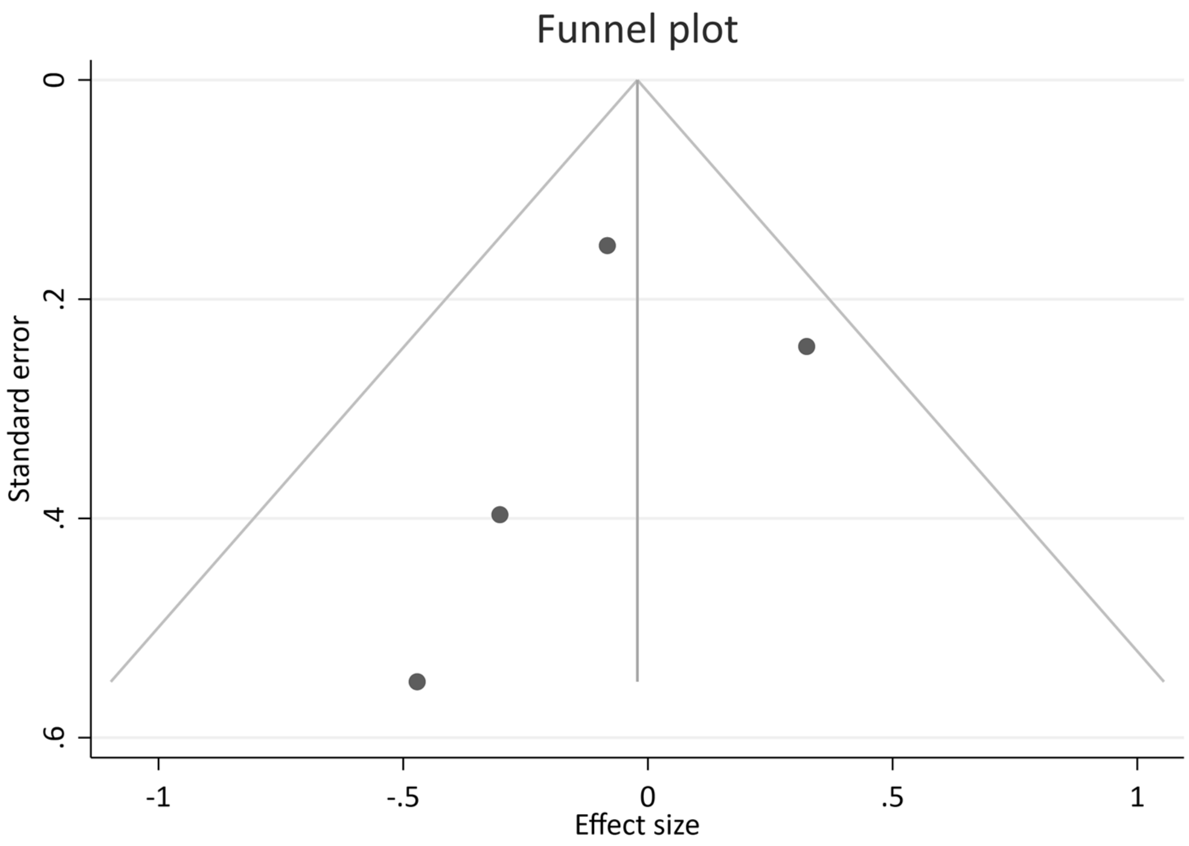

Figure 19: Funnel plot for PFC (AUC).

Supplementary table 19 – Risk of bias assessment

	Non-randomized trials							Randomized control trials					
**Author (Year)**	**1. Bias due to confounding**	**2. Bias in selection of participants into the study**	**3. Bias in classification of interventions**	**4. Bias due to deviations from intended interventions**	**5. Bias due to missing data**	**6. Bias in measurement of the outcome**	**7. Bias in selection of the reported result**	**1. Bias arising from the randomization process;**	**2. Bias due to deviations from intended interventions;**	**3. Bias due to missing outcome data;**	**4. Bias in measurement of the outcome**	**5. Bias in selection of the reported result**	**6. Overall**
**Acosta (2015)**	**low**	**moderate**	**low**	**moderate**	**moderate**	**low**	**low**						
**Adam (2005)**								**low**	**low**	**NA**	**moderate**	**low**	**moderate**
**Andarini (2017)**	**low**	**low**	**low**	**low**	**NA**	**moderate/high**	**low**						
**Arafat (2013)**								**low**	**low**	**NA**	**low**	**low**	**low**
**Barkeling (1995)**	**low**	**moderate**	**low**	**low**	**NA**	**moderate**	**low**						
**Batterham (2003)**								**low**	**low**	**NA**	**low**	**low**	**low**
**Bogdanov (2019)**	**low**	**low**	**low**	**moderate**	**moderate**	**moderate**	**low**						
**Bowen (2006)**								**low**	**low**	**NA**	**low**	**low**	**low**

**Brownley (2010)**	**moderate**	**moderate**	**low**	**low**	**moderate**	**low**	**low**						
**Brownley (2012)**	**low**	**moderate**	**low**	**moderate**	**NA**	**low**	**low**						
**Cahill (2011)**	**low**	**low**	**low**	**low**	**NA**	**moderate**	**low**						
**Cahill (2014)**	**low**	**low**	**low**	**low**	**moderate**	**moderate**	**low**						
**Calanna (2013)**	**moderate**	**low**	**low**	**low**	**NA**	**moderate**	**low**						
**Carlson (2009)**	**low**	**moderate**	**low**	**moderate**	**NA**	**low**	**low**						
**Carr (2010)**	**low**	**moderate**	**low**	**low**	**NA**	**low**	**low**						
**Carroll (2007)**	**moderate**	**low**	**low**	**moderate**	**NA**	**low**	**low**						
**Cassar (2015)**								**low**	**low**	**NA**	**low**	**low**	**low**
**Chearskul (2012)**	**low**	**low**	**low**	**low**	**NA**	**low**	**low**						
**Chia (2017)**								**low**	**low**	**NA**	**moderate**	**low**	**moderate**
**Clamp (2015)**	**low**	**low**	**low**	**low**	**NA**	**low**	**low**						
**Cremonini (2006)**								**low**	**low**	**moderate**	**low**	**low**	**moderate**
**Daghestani (2009)**	**moderate**	**moderate**	**low**	**low**	**NA**	**moderate**	**low**						
**Damgaard (2013)**								**low**	**low**	**NA**	**low**	**low**	**low**
**Dardzinska (2014)**	**low**	**low**	**low**	**low**	**NA**	**moderate**	**low**						
**DeBenedictis (2020)**	**low**	**moderate**	**low**	**low**	**NA**	**moderate**	**low**						
**Dirksen (2019)**								**low**	**moderate**	**NA**	**low**	**low**	**moderate**

**Douglas (2017)**								**low**	**low**	**NA**	**modertae**	**low**	**moderate**
**Druce (2005)**								**moderate**	**low**	**NA**	**low**	**low**	**moderate**
**El Helou (2019)**								**low**	**low**	**NA**	**moderate**	**low**	**moderate**
**Elahi (2016)**	**moderate**	**low**	**low**	**low**	**NA**	**modertae**	**low**						
**English (2002)**	**moderate**	**low**	**low**	**low**	**NA**	**low**	**low**						
**Erdmann (2005)**	**low**	**low**	**low**	**low**	**NA**	**low**	**low**						
**Espelund (2005)**	**low**	**moderate**	**low**	**low**	**NA**	**low**	**low**						
**Frecka (2008)**	**low**	**low**	**low**	**low**	**NA**	**low**	**low**						
**French (1993)**								**low**	**low**	**NA**	**low**	**low**	**low**
**Greenfield (2009)**								**low**	**low**	**NA**	**low**	**low**	**low**
**Guo (2007)**	**low**	**low**	**low**	**low**	**NA**	**low**	**low**						
**Haltia (2010)**								**moderate**	**moderate**	**NA**	**low**	**low**	**moderate**
**Heden (2013)**								**low**	**low**	**NA**	**moderate**	**low**	**moderate**
**Heden (2013)**								**low**	**low**	**NA**	**modertae**	**loe**	**moderate**
**Heni (2015)**	**low**	**moderate**	**low**	**low**	**NA**	**moderate**	**low**						
**Homaee (2011)**	**low**	**moderate**	**low**	**low**	**NA**	**moderate**	**low**						
**Iceta (2019)**	**low**	**moderate**	**low**	**low**	**low**	**moderate**	**low**						
**Karcz-Socha et al. 2011**	**low**	**low**	**low**	**low**	**NA**	**low**	**low**						

**Kheirouri et al. 2017**								**low**	**moderate**	**NA**	**moderate**	**low**	**moderate**
**Kiessl et al. 2017**								**low**	**moderate**	**NA**	**moderate**	**low**	**moderate**
**Kocak et al. 2011**	**low**	**moderate**	**low**	**low**	**NA**	**moderate**	**low**						
**Kolodziejski et al. 2018**	**low**	**moderate**	**low**	**low**	**NA**	**low**	**low**						
**Korek et al. 2013**	**low**	**low**	**low**	**low**	**NA**	**low**	**low**						
**Korner et al. 2005**	**low**	**moderate**	**low**	**low**	**low**	**low**	**low**						
**Krzyzanowska-Swinirska (2007)**	**low**	**moderate**	**low**	**low**	**NA**	**low**	**low**						
**Lambert et al. 2011**								**low**	**moderate**	**NA**	**high**	**low**	**moderate**
**Leonetti et al.2003**	**low**	**low**	**low**	**low**	**NA**	**low**	**low**						
**Lieverse et al. 1993**								**low**	**low**	**NA**	**low**	**low**	**low**
**Lieverse et al. 1998**								**low**	**low**	**NA**	**low**	**low**	**low**
**Lopez-Aguilar et al. 2018**	**low**	**moderate**	**low**	**low**	**NA**	**moderate**	**low**						
**Marzullo et al. 2004**	**moderate**	**low**	**low**	**low**	**NA**	**low**	**low**						
**Marzullo et al. 2006**	**low**	**low**	**low**	**low**	**low**	**low**	**low**						
**Mersebach et al. 2003**	**low**	**low**	**low**	**low**	**NA**	**low**	**low**						
**Meyer-Gerspach et al. 2014**	**low**	**moderate**	**low**	**low**	**moderate**	**moderate**	**low**						
**Milewicz et al. 2000**	**low**	**moderate**	**low**	**low**	**NA**	**low**	**low**						
**Nguyen et al. 2018**	**low**	**moderate**	**low**	**low**	**NA**	**low**	**low**						

**Outeiriño-Blanco et al. 2011**	**low**	**moderate**	**low**	**low**	**NA**	**low**	**low**						
**Ozkan et al. 2009**								**low**	**low**	**NA**	**low**	**low**	**low**
**Painchaud Guerard et al. 2016**								**low**	**moderate**	**moderate**	**low**	**low**	**moderate**
**Papandreou et al. 2017**	**low**	**low**	**low**	**low**	**NA**	**moderate**	**low**						
**Pavlatos et al. 2005**								**low**	**low**	**NA**	**low**	**low**	**low**
**Pfluger et al. 2007**								**low**	**low**	**NA**	**moderate**	**low**	**moderate**
**Smith et al. (2021)**								**low**	**low**	**NA**	**moderate**	**low**	**moderate**
**Verdich et al. (2001)**	**low**	**low**	**low**	**moderate**	**NA**	**low**	**low**						

NA= not assessed/applicable

Supplementary table 20. Study characteristics

Study	Participants CO/OB	Test meal composition	Postprandial period	Hormone measure	Basal hormone concentrations CO vs OB	Postprandial hormone response CO vs OB (AUC)	Self-reported appetite ratings CO vs OB (VAS)
Acosta (2015) # (14)	BMI (kg/m^2): 27.6 ± 0.1 / 35.5 ± 3.9<br>Age: 37.3 ± 12.0 / 37.8 ± 12.1<br>N (F%): 105 (61%) / 201 (72%)			Total ghrelin [a] *	No difference<br>P=0.063		
Adam (2005) (94)	BMI: 22.9 ± 1.5 / 30.4 ± 2.7<br>Age: 31.6 ± 12.8 / 44.4 ± 9.8<br>N (F%): 30 (50%) / 28 (68%)	454 kcal<br>Protein (28.5%)<br>CHO (48.8%)<br>Fat (22.6%)<br>Fiber (NA)	120 minutes<br>0, 30, 60, 90, 120	Active GLP-1[b] *	No difference<br>P>0.05	CO > OB<br>P=0.03	No differences in fullness or DTE for both fasting and postprandial (P>0.05)
Andarini (2017) (47)	BMI: 21.3 ± 1.1 / 33.6 ± 4.8<br>Age: 20.6 ± 1.1 / 21.4 ± 1.9<br>N (F%): 16 (0%) / 16 (0%)	570 kcal<br>Protein (3.2%)<br>CHO (14.8%)<br>Fat (4.6%)<br>Fiber (5.9 g)	120 minutes<br>0, 30, 60, 120	Active ghrelin [c]*	CO < OB<br>P<0.01	CO < OB<br>P<0.01	No differences in hunger or DTE for both fasting and postprandial (P>0.05)*
Arafat (2013) (26)	BMI: 21.7 ± 2.2 / 34.4 ± 5.6<br>Age: 25.1 ± 2.2 / 28.4 ± 8.6<br>N (F%): 13 (54%) / 11 (55%)			Active ghrelin [a]*<br>Total ghrelin [a]*	CO > OB<br>P<0.01, for both		
Barkeling (1995) # (132)	BMI: 22.3 ± 1.9 / 39.4 ± 6.5<br>Age: 40.0 ± 10.9 / 43.5 ± 12.0<br>N (F%): 38 (50%) / 38 (50%)						Fasting hunger, DTE, and PFC:<br>CO>OB (P<0.0001) *<br>Fasting fullness:<br>CO<OB (P<0.0001) *
Batterham (2003) (49)	BMI: 20.5 ± 0.3 / 33.0 ± 3.1<br>Age: 27.3 ± 1.4 / 29.0 ± 8.3<br>N (F%): 12 (50%) / 12 (50%)			PYY 3-36 [d] *<br>Total ghrelin [e]*	PYY CO > OB<br>P<0.001<br>Ghrelin CO > OB<br>P<0.001		
Bogdanov (2019) (51)	BMI: 21.8 ± 2.6 / 41.2 ± 2.1<br>Age: 37.0 ± 7.1 / 38.7 ± 9.4<br>N (F%): 15 (87%) / 15 (80%)			Total ghrelin [f]*	CO > OB<br>P<0.001		
Bowen (2006) (52)	BMI: 23.3 ± 1.0 / 30.1 ± 3.4<br>Age: 50.5 ± 12.0 / 56.8 ± 7.5			Total ghrelin [g]*<br>Active GLP-1 [h]*	Ghrelin CO > OB<br>P<0.001		

	N (F%): 25 (0%) / 47 (0%)			CCK [i*]	GLP-1 CO < OB<br>P<0.001<br>CCK no difference<br>P>0.05	
Brownley (2010) # (120)	BMI: 22.9 ± 1.4 / 34.7 ± 2.8<br>Age: 27.9 ± 7.0 / 34.9 ± 8.9<br>N (F%): 20 (50%) / 20 (50%)	625 kcal<br>Protein (14.7%)<br>CHO (55.0%)<br>Fat (30.2%)<br>Fiber (NA)	180 minutes<br>0, 30, 60, 120, 180	Total PYY [a*]		
Brownley (2012) # (53)	BMI: 22.9 ± 1.4 / 34.7 ± 2.8<br>Age: 27.9 ± 7.0 / 34.9 ± 8.9<br>N (F%): 20 (50%) / 20 (50%)	625 kcal<br>Protein (14.7%)<br>CHO (55.0%)<br>Fat (30.2%)<br>Fiber (NA)	180 minutes<br>-20, 30, 60, 90, 105, 120, 135, 150, 180	Total ghrelin [a*]	CO>OB<br>P<0.001	CO> OB<br>P<0.05
Cahill (2011) # (124)	BMI: 23.1 ± 2.3 / 29.1 ± 4.9<br>Age: 23.1 ± 3.5 / 23.2 ± 2.6<br>N (F%): 41 (0%) / 28 (0%)			Total PYY [f*]	No difference<br>P>0.05	
Cahill (2014) # (122)	BMI: 24.4 ± 3.1 / 30.6 ± 4.8<br>Age: 42.2 ± 13.2 / 43.8 ± 12.1<br>N (F%): 1315 (76%) / 779 (74%)			Total PYY [f*]	No difference<br>P>0.05	
Calanna (2013) (95)	BMI: 22.1 ± 2.0 / 34.6 ± 3.9<br>Age: 38.3 ± 9.8 / 42.8 ± 13.1<br>N (F%): 24 (83%) / 43 (67%)			Active GLP-1 [f*]	No difference<br>P>0.05	
Carlson (2009) (54)	BMI: 23.1 ± 1.3 / 44.5 ± 7.1<br>Age: 32.2 ± 8.6 / 35.6 ± 9.7<br>N (F%): 10 (100%) / 13 (100%)			Total ghrelin [a*]	CO > OB<br>P=0.001	
Carr (2010) (105)	BMI: 22.3 ± 0.3 / 33.8 ± 0.6<br>Age: 22.0 ± 1.8 / 25.6 ± 4.0<br>N (F%): 12 (0%) / 13 (0%)	560 kcal<br>Macronutrient content of meal not specified.	300 minutes<br>0, 30, 45, 60, 90, 120, 150, 180, 240, 300	Total GLP-1 [j*]	No difference<br>P>0.05	CO > OB<br>P=0.022
Carroll (2007) # (40)	BMI: 22.6 ± 1.3 / 36.8 ± 4.8<br>Age: 39.2 ± 10.3 / 47.2 ± 10.9	379 kcal (528 ml)<br>Protein (25.4 %)	60 minutes	Active ghrelin [a*]<br>Active GLP-1 [a*]	No differences in ghrelin or GLP-1.	No differences in ghrelin or GLP-1.

	N (F%): 19 (47%) / 20 (55%)	CHO (42.3 %)<br>Fat (7.6 %)<br>Fiber (15.0g)	0, 10, 20, 30, 40, 50, 60		P>0.05	P>0.05	
Cassar (2015) (55)	BMI: 22.0 ± 2.0 / 31.0 ± 3.0<br>Age: 28.0 ± 6.0 / 35.0 ± 5.0<br>N (F%): 22 (100%) / 18 (100%)			Total ghrelin [k]<br>Total GLP-1 [k]	Ghrelin CO > OB<br>P<0.05.<br>No difference in GLP-1<br>P>0.05		
Chearskul (2012) (41)	BMI: 20.7 ± 2.2 / 29.4 ± 3.6<br>Age: 30.1 ± 5.8 / 33.6 ± 6.3<br>N (F%): 53 (100%) / 33 (100%)			Active ghrelin [a*]	No difference<br>P>0.05		
Chia (2017) (106)	BMI: 23.6 ± 1.3 / 35.6 ± 4.0<br>Age: 68.5 ± 13.0 / 68.5 ± 13.0<br>N (F%): 20 (50%) / 20 (50%)			Total GLP-1 [l*]	No difference<br>P>0.05		
Clamp (2015) (84)	BMI: 22.3 ± 1.6 / 33.2 ± 3.0<br>Age: 25.0 ± 5.0 / 30.0 ± 6.0<br>N (F%): 10 (0%) / 10 (0%)			Total ghrelin [g*]<br>Total PYY [a*]	No differences in ghrelin and PYY<br>P>0.05		
Cremonini (2006) (85)	BMI: 22.0 ± 2.0 / 36.0 ± 4.0<br>Age: 34.0 ± 12.0 / 35.0 ± 8.0<br>N (F%): 13 (100%) / 25 (80%)			Total ghrelin [a*]	No difference<br>P=0.068		
Daghestani (2009) # (57)	BMI: 22.3 ± 3.3 / 35.9 ± 6.2<br>Age: 23.5 ± 4.5 / 26.5 ± 6.4<br>N (F%): 77 (100%) / 45 (100%)	527 kcal<br>Protein 21.5%<br>CHO 54.4%<br>Fat 24.1%<br>Fiber (NA)		Total ghrelin [m*]	CO > OB<br>P<0.001		
Damgaard (2013) # (107)	BMI: 22.1 ± 2.3 / 30.6 ± 3.5<br>Age: 27.1 ± 6.1 / 32.3 ± 7.7<br>N (F%): 8 (0%) / 13 (0%)		240 minutes<br>Blood collection: 0, 7.5, 15, 22.5, 30, 37.5, 45, 52.5, 60, 90, 120, 150, 180, 210, 240<br>VAS: 0, 30, 60, 90, 120, 150, 180, 210, 240	Total GLP-1 [d]<br>CCK [d]<br>PYY 3-36 [n*]	No difference in GLP-1 or CCK<br>P>0.05<br>No difference in PYY<br>P= 0.15*		No differences in fasting hunger or postprandial hunger or fullness<br>P>0.05 *

Dardzinska (2014) (27)	BMI: 23.0 ± 3.5 / 43.8 ± 6.8<br>Age: 37.2 ± 9.4 / 35.4 ± 9.1<br>N (F%): 12 (92%) / 12 (71%)	300 kcal (200 ml)<br>Protein 16.0%<br>CHO 49.0%<br>Fat 35.0%<br>Fiber (NA)	120 minutes<br>Intervals not given	Active ghrelin [o*]	CO > OB<br>P<0.00001	Ghrelin CO > OB<br>P<0.05	
DeBenedictis (2020) (16)	BMI: 24.8 ± 0.4 / 34.0 ± 0.4<br>Age: 38.9 ± 2.0 / 45.0 ± 1.5<br>N (F%): 33 (49%) / 34 (50%)	600 kcal<br>Protein (17%)<br>CHO (48%)<br>Fat (35%)	150 minutes<br>Intervals not given	Active ghrelin [ø*]<br>Total GLP-1 [r*]<br>Total PYY [ø*]<br>CCK [r*]		Ghrelin, GLP-1, PYY and CCK<br>CO > OB<br>P<0.001, for all	No differences in fasting or postprandial hunger, fullness, DTE or PFC *<br>P > 0.05
Dirksen (2019) # (104)	BMI: 24.1 ± 3.8 / 57.6 ± 17.2<br>Age: 43.1 ± 8.9 / 42.1 ± 9.8<br>N (F%): 10 (0%) / 10 (0%)			Total GLP-1 [d*]<br>PYY 3-36 [n*]<br>CCK [d*]	GLP-1: CO > OB<br>P<0.05<br>No differences in PYY or CCK<br>P>0.05		No differences in fasting hunger or fullness<br>P>0.05, for both*
Douglas (2017) (42)	BMI: 22.4 ± 1.5 / 29.2 ± 2.9<br>Age: 37.5 ± 15.2 / 45.0 ± 12.4<br>N (F%): 22 (44%) / 25 (50%)	610 kcal<br>Protein 10.0 %<br>CHO 72.0 %<br>Fat 18.0 %<br>Fiber (NA)		Active ghrelin [p*]<br>Total GLP-1 [f*]<br>Total PYY [f*]	No differences in ghrelin, GLP-1, or in PYY<br>P>0.05, for all		No differences in hunger, fullness, or PFC (both in fasting and postprandially)<br>P>0.05, for all
Druce (2005) (86)	BMI: 20.5 ± 0.6 / 31.9 ± 3.5<br>Age: 24.7 ± 4.6 / 33.4 ± 6.9<br>N (F%): 12 (50%) / 12 (50%)			Total Ghrelin [d*]	No difference.<br>P>0.05		
El Helou (2019) (58)	BMI: 22.0 ± 1.9 / 35.1 ± 3.9<br>Age: 20.1 ± 1.5 / 21.7 ± 3.5<br>N (F%): 15 (0%) / 15 (0%)		240 minutes<br>0, 15, 30, 60, 120, 180, 240	Total ghrelin [q*]<br>Total GLP-1 [f*]	Ghrelin: CO > OB<br>P=0.050<br>GLP-1: No difference<br>P>0.05		No differences in fasting hunger, fullness, or PFC<br>P>0.05
Elahi (2016) (96)	BMI: 22.3 ± 1.4 / 37.2 ± 5.2<br>Age: 29.0 ± 6.4 / 42.0 ± 6.9<br>N (F%): 12 (50%) / 12 (50%)			Total GLP-1 [b*]<br>Active GLP-1 [b]	total GLP-1 not compared<br>No difference in active GLP-1		
English (2002) (28)	BMI: 22.5 ± 1.9 / 42.8 ± 6.4<br>Age: 32.0 ± 8.3 / 42.8 ± 7.0<br>N (F%): 13 (60%) / 10 (30%)	632 kcal<br>Protein 12.2%<br>CHO 56.8%	180 minutes<br>0, 15, 30, 60, 120, 180	Total ghrelin [d*]	CO > OB<br>P=0.002		

		Fat 31.0%<br>Fiber (NA)					
Erdmann (2005) (59)	BMI: 22.0 ± 2.2 / 33.8 ± 5.7<br>Age: 30.0 ± 10.5 / 45.0 ± 13.6<br>N (F%): 56 (80%) / 128 (36%)	260 kcal<br>Protein 6.0%<br>CHO 62.0%<br>Fat 32.0%<br>Fiber (NA)	180 minutes<br>-15, 0, 15, 30, 60, 90, 120, 150, 180	Total ghrelin [g*]	CO > OB<br>P<0.01		
Espelund (2005) (87)	BMI: 23.4 ± 2.5 / 29.5 ± 4.0<br>Age: 33.7 ± 14.0 / 39.7 ± 12.4<br>N (F%): 17 (47%) / 16 (56%)			Total ghrelin [r*]	No difference<br>P>0.05		
Frecka (2008) (88)	BMI: 23.3 ± 1.6 / 32.2 ± 1.6<br>Age: 24.3 ± 4.2 / 32.0 ± 9.6<br>N (F%): 7 (57%) / 5 (40%)	501 ± 218 kcal<br>Protein (11.1%)<br>CHO (47.2%)<br>Fat (29.7%)	330 minutes<br>Intervals not given	Total ghrelin [a*]	No difference<br>P>0.05	No difference<br>P>0.05	No differences in fasting hunger, fullness, DTE, or PFC<br>P>0.05, for all
French (1993) (18)	BMI: (20-25) / (<30)<br>Age: (22-44) / (21-48)<br>N (F%): 7 (57%) / 8 (63%)	317 kcal<br>Macronutrient content of meal not specified	180 minutes<br>0, 10, 20, 30, 60, 90, 120, 150, 180	CCK [d*]	CO < OB<br>P<0.01	CO < OB<br>P<0.05	No differences in fasting hunger, fullness or DTE, P>0.05, for all.<br>Postprandial hunger CO > OB P<0.05.<br>No differences in postprandial fullness or DTE<br>P>0.05, for both
Greenfield (2009) (108)	BMI: 21.9 ± 2.2 / 34.5 ± 4.4<br>Age: 30.0 ± 5.8 / 39.0 ± 9.8<br>N (F%): 7 (57%) / 8 (63%)			Total GLP-1 [d*]	No difference<br>P>0.05		
Guo (2007) (60)	BMI: 21.6 ± 1.7 / 30.1 ± 1.9<br>Age: 54.4 ± 5.4 / 59.4 ± 7.3<br>N (F%): 16 (50%) / 14 (50%)	500 - 600 kcal<br>Macronutrient content of meal not specified	Blood collection at 0 minutes	Total ghrelin [g*]	CO > OB<br>P<0.01		
Haltia (2010) (89)	BMI: 21.7 ± 1.3 / 33.0 ± 4.5<br>Age: 26.0 ± 5.0 / 27.0 ± 6.0<br>N (F%): 12 (50%) / 13 (38%)			Total ghrelin [a*]<br>PYY 3-36 [a*]<br>Total GLP-1 [b*]	No differences in ghrelin, PYY or GLP-1<br>P>0.05, for all		

Heden (2013) (97)	BMI: 23.0 ± 1.8 / 34.6 ± 3.6<br>Age: 26.0 ± 7.2 / 25.4 ± 3.6<br>N (F%): 13 (46%) / 13 (54%)	600 kcal<br>Protein (15%)<br>CHO (45%)<br>Fat (40%)	240 minutes<br>0, 5, 10, 15, 20, 30, 40, 50, 60, 75, 90, 120, 150, 180, 210, 240	Active GLP-1 [s*]		No difference<br>P>0.05	
Heden (2013) (29)	BMI: 22.9 ± 1.7 / 34.8 ± 4.4<br>Age: 26.0 ± 6.0 / 25.1 ± 5.0<br>N (F%): 14 (43%) / 14 (57%)	600 kcal<br>Protein (15%)<br>CHO (45%)<br>Fat (40%)	Blood collection at 0 minutes	Active ghrelin [s*]	No difference<br>P=0.06		No differences in fasting hunger and fullness<br>P>0.05, for both
Heni (2015) (109)	BMI: 21.2 ± 3.8 / 30.5 ± 6.2<br>Age: 23.0 ± 6.9 / 25.0 ± 6.9<br>N (F%): 12 (50%) / 12 (50%)			Total GLP-1 [t*]	No difference<br>P>0.05		
Homaee (2011) (30)	BMI: 18.5 ± 2.2 / 31.0 ± 3.6<br>Age: 26.9 ± 5.6 / 27.5 ± 5.8<br>N (F%): 19 (0%) / 19 (0%)			Active ghrelin [u*]	CO > OB<br>P=0.008		
Iceta (2019) (31)	BMI: 21.5 ± 2.2 / 41.5 ± 5.9<br>Age: 37.0 ± 10.8 / 38.0 ± 11.1<br>N (F%): 29 (100%) / 55 (100%)			Active ghrelin [o*]	CO > OB<br>P<0.001		No differences in fasting hunger<br>P>0.05
Karcz-Socha (2011) # (45)	BMI: 23.4 ± 1.5 / 35.7 ± 2.9<br>Age: 51.2 ± 6.5 / 51.5 ± 6.5<br>N (F%): 46 (52%) / 96 (50%)			Active ghrelin [a*]<br>Total ghrelin [a*]<br>PYY 3-36 [a*]	No difference in acylated ghrelin.<br>Total ghrelin CO > OB<br>P<0.001<br>PYY CO > OB<br>P<0.001		
Kheirouri (2017) (62)	BMI: 23.1 ± 0.8 / 31.4 ± 0.7<br>Age: 35.2 ± 7.9 / 37.2 ± 7.5<br>N (F%): 40 (100%) / 37 (100%)			Total ghrelin [v*]	CO > OB<br>P = 0.03		
Kiessl (2017) (63)	BMI: 21.7 ± 2.0 / 31.5 ± 1.8<br>Age: 18-30 / 18-30<br>N (F%): 42 (100%) / 43 (100%)	500 g pudding, eat as much as wanted.<br>per100 g 158 kcal	60 minutes<br>0, 30, 60	Total ghrelin [f*]	CO > OB<br>P<0.001	CO > OB<br>P<0.001	
Kocak (2011) (64)	BMI: 25.2 ± 1.7 / 34.1 ± 4.0<br>Age: 58.6 ± 10.3 / 53.0 ± 8.5<br>N (F%): 19 (100%) / 22 (100%)			Total ghrelin [w*]	CO > OB<br>P<0.001		

Kolodziejski (2018) (43)	BMI: 22.3 ± 0.5 / 39.8 ± 1.0<br>Age: 42.9 ± 5.3 / 42.2 ± 3.3<br>N (F%): 15 (100%) / 15 (100%)			Active ghrelin [n*]<br>Total ghrelin [n*]<br>Total GLP-1 [m]	Acylated ghrelin:<br>CO < OB<br>P<0.01<br>No difference for total ghrelin or GLP-1<br>P>0.05	
Korek (2013) (32)	BMI: 21.1 ± 1.85 / 34.7 ± 4.92<br>Age: 19-35 / 20-35<br>N (F%): 17 (89%) / 19 (90%)	260 kcal<br>Protein (8%)<br>CHO (43%)<br>Fat (49%)	Blood collection at 0 and 120 minutes after breakfast	Active ghrelin [a*]<br>Total ghrelin [n*]	CO > OB<br>P<0.05	
Korner (2005) (90)	BMI: 21.6 ± 0.7 / 34.1 ± 1.8<br>Age: 30.6 ± 3.6 / 44.3 ± 4.3<br>N (F%): 8 (100%) / 12 (100%)	320 kcal<br>Protein (35%)<br>CHO (50%)<br>Fat (15%)		Total ghrelin [g*]<br>PYY 3-36 [g*]	No difference<br>Ghrelin, P = 0.1<br>PYY, P>0.05	No differences in fasting hunger or fullness<br>P>0.05
Krzyzanowska-Swinirska (2007) (46)	BMI: 21.3 ± 1.7 / 34.4 ± 4.1<br>Age: 28.8 ± 4.8 / 32.5 ± 6.5<br>N (F%): 32 (100%) / 30 (100%)			Active ghrelin [a*]	No difference	
Lambert (2011) # (91)	BMI: 21.3 ± 0.6 / 29.3 ± 0.6<br>Age: 21.5 ± 0.5 / 20.8 ± 0.6<br>N (F%): 11 (0%) / 11 (0%)			Total ghrelin [x*]	No difference	
Leonetti (2003) (65)	BMI: 23.0 ± 2.5 / 35.9 ± 3.6<br>Age: 40.2 ± 10.6 / 41.2 ± 11.6<br>N (F%): 10 (50%) / 8 (70%)			Total ghrelin [g*]	CO > OB<br>P<0.01	
Lieverse (1993) (126)	BMI: 22.3 ± 2.1/ 40.7 ± 6.6<br>Age: 41.2 ± 11.6 / 40.2 ± 10.6<br>N (F%): 7 (100%) / 7 (100%)			CCK [d*]	No difference	
Lieverse (1998) (125)	BMI: 39 ± 2.0 / 22 ± 0.3<br>Age: 43 ± 3 / 42 ± 3<br>N (F%): 7 (100%) / 7 (100%)			CCK [d*]	CO > OB<br>P<0.05	

Lopez-Aguilar (2018) (33)	BMI: 22.7 ± 1.5 / 35.4 ± 5.2<br>Age: 26.4 ± 5.6 / 29.2 ± 6.3<br>N (F%): 80 (67%) / 50 (52%)			Active ghrelin [v*]	CO > OB<br>P=0.009	
Marzullo (2004) (35)	BMI: 22.4 ± 0.6 / 41.3 ± 1.1<br>Age: 31.7 ± 1.3 / 32.4 ± 1.6<br>N (F%): 20 (50%) / 20 (50%)			Active ghrelin [a*]<br>Total ghrelin [g]	CO > OB<br>P<0.05	
Marzullo (2006) (17)	BMI: 21.8 ± 1.4 / 43 ± 0.9<br>Age: 33.5 ± 2.4 / 31.8 ± 2.5<br>N (F%): 6 (50%) / 10 (50%)	500 kcal<br>Liquid meal<br>Proteins (17%)<br>CHO (53%)<br>Fat (30%)	120 minutes<br>0, 20, 40, 60, 80, 100, 120	Total ghrelin [g*]		CO > OB<br>P<0.01
Mersebach (2003) (98)	BMI: 21.5 ± 1.3 / 36.1 ± 2.4<br>Age: 37.9 ± 5.9 / 39.3 ± 3.5<br>N (F%): 10 (70%) / 16 (75%)			Active GLP-1 [d*]	No difference	
Meyer-Gerspach (2014) (15)	BMI: 21.8 ± 0.4 / 39.3 ± 1.9<br>Age: 24.1 ± 0.6 / 29.8 ± 1.9<br>N (F%): 20 (NA%) / 20 (NA%)	750 kcal (500ml)<br>Liquid meal<br>Protein (17%)<br>CHO (54%)<br>Fat (29%)	180 minutes<br>−1, 30, 60, 120, 180	Total ghrelin [a*]<br>Active GLP-1 [f*]<br>Total PYY [a*]	Ghrelin: CO > OB<br>P = 0.027	Ghrelin: CO > OB<br>P = 0.001<br>GLP-1: CO > OB<br>P<0.001<br>PYY: CO > OB<br>P<0.001
Milewicz (2000) # (127)	BMI: 21.2 ± 0.4 / 34.6 ± 1.4<br>Age: NA / NA<br>N (F%): 16 (NA%) / 25 (NA%)			CCK [y*]	No difference	
Nguyen (2018) (110)	BMI: 23.9 ± 0.7 / 48.6 ± 1.8<br>Age: 38.6 ± 8.4 / 50.2 ± 2.5<br>N (F%): 10 (59%) / 22 (50%)	302 kcal<br>50 g minced beef<br>150 ml dextrose solution<br>75 g glucose loading	240 minutes<br>−2, 15, 30, 45, 60, 75, 90, 120, 150, 180, 210, 240	Total GLP-1 [d*]	No difference	
Outeiriño-Blanco (2011) (67)	BMI: 22.3 ± 0.7 / 38.8 ± 1.2<br>Age: 34.4 ± 3.6 / 39.8 ± 2.9<br>N (F%): 13 (100%) / 23 (100%)	75 g glucose loading	150 minutes<br>0, 30, 60, 90, 120, 150	Total ghrelin [a*]<br>PYY 1-36 [a*]	Ghrelin: CO > OB<br>P= 0.01<br>PYY: no difference	Ghrelin: CO > OB<br>P= 0.026<br>PYY: no difference

Ozkan (2009) (68)	BMI: 24.8 ± 3.1 / 37 ± 3.6<br>Age: 36.1 ± 9.8 / 37.4 ± 12.4<br>N (F%): 10 (60%) / 21 (71%)			Total ghrelin [z*]	CO > OB<br>P= 0.001		
Painchaud Guerard (2016) # (135)	BMI: 24.2 ± 2.9 / 33.15 ± 3.6<br>Age: 36.7 ± 14.7 / 44.2 ±16.2<br>N (F%): 302 (53%) / 51 (55%)	Ad libitum oatmeal raisin snack	60 minutes<br>-10, 0, 20, 40, 60				No differences in fasting or postprandial hunger, fullness, DTE or PFC *
Papandreou (2017) (69)	BMI: 20.6 ± 3 / 31.3 ± 2.1<br>Age: 21 ± 0.8 / 21 ± 2.07<br>N (F%): 13 (NA%) / 7 (NA%)			Total ghrelin [w*]	CO > OB<br>P= 0.002		
Pavlatos (2005) (70)	BMI: 22.9 ± 2.1 / 37.2 ± 8.4<br>Age: 38.7 ± 14.1 / 39.5 ± 14.5<br>N (F%): 9 (100%) / 9 (100%)			Total ghrelin [a*]	CO > OB<br>P= 0.001		
Pfluger (2007) # (117)	BMI: 22.1 ± 0.2 / 35.1 ± 4.8<br>Age: 41.5 ± 2.2 / 47.7 ± 2.9<br>N (F%): 66 (70%) / 79 (73%)			Total PYY [æ*]	No difference		
Pfluger (2007) # (117)	BMI: 22.0 ± 0.5 / 31.1 ± 0.5<br>Age: 51.6 ± 1.9 / 52.0 ± 2.0<br>N (F%): 17 (100%) / 15 (100%)			PYY 3-36 [a*]	No difference		
Smith (2021) # (103)	BMI: 23.7 ± 1.8 / 33.7 ± 2.4<br>Age: 35.8 ± 10.6 / 34.8 ± 7.4<br>N (F%): 12 (0%) / 12 (0%)	387 kcal<br>Protein (15%)<br>CHO (58%)<br>Fat (27%)	240 minutes	Active GLP-1 [å*]<br>Total GLP-1 [å*]	No difference in active GLP-1<br>Total GLP-1:<br>CO<OB<br>P <0.05		No differences in postprandial hunger, fullness, or PFC*
Verdich (2001) (113)	BMI: 23.1 ± 1.4 / 38.1 ± 3.1<br>Age: 24.2 ± 9.6 / 35.0 ± 10.9<br>N (F%): 12 (0%) / 19 (0%)	597 kcal<br>Protein (20%)<br>CHO (50%)<br>Fat (30%)	180 minutes<br>0, 20, 40, 60, 80, 100, 120, 140, 160, 180	Total GLP-1[d*]		CO > OB<br>P<0.01	

AUC: area under the curve. BMI: body mass index. CCK: cholecystokinin. CHO: carbohydrate. CO: control. DTE: desire to eat. GLP-1: glucagon-like peptide 1. NA: not assessed/compared. OB: obesity. PFC: prospective food consumption. PYY: peptide YY. VAS: visual analogue scale. Superscript letters denote hormone analyses method used: [a]RIA (Linco Research, Inc., St. Charles, MO). [b]ELISA (Linco, St. Charles, MO, USA). [c]ELISA (Elabscience, Biotechnology, Beijing). [d]RIA manufacturer not specified. [e] Hormone assay not specified. [f]ELISA (Millipore Corporation Pharmaceuticals, Billerica, MA). [g]RIA (Phoenix Pharmaceuticals, Belmont, CA). [h]Fluorescence immunoassay (Linco). [i]RIA (Euria-Diagnostica, Malmo, Sweden). [j]Amino terminal-specific assay (Linco Research). [k]Bio-Plex Pro Diabetes assay (CAT#171-A7001M; Biorad Laboratories, Hercules, CA, USA). [l]ELISA (Alpco Diagnostics, Salem, NH). [m]ELISA (Phoenix Pharma-ceuticals, INc (Belmont, CA)). [n]RIA (Millipore, Billerica, MA, USA). [o]Human Acylated Ghrelin EIA Kit (Biovendor, Czech Republic). [p]ELISA (SPI BIO, Montigny le Bretonneux, France). [q]ELISA (EMD Millipore Corp., St. Charles, Missouri). [r]«inhouse » RIA. [s]MILLIPLEX magnetic bead-based quantitative multiplex immunoassay with the MAGPIX instrumentation (Millipore, Billerica, MA). [t]ELISA (Millipore, Watford, UK). [u]ELISA (Acylated ghrelin Human ELISA, BioVendor, Germany. [v]ELISA, inspecified. [w]Immunochemilunometric assay, (IDS, SMBH, Germany). [x]ELISA (Phoenix Pharmaceuticals, Inc, Burlingame, CA). [y]RIA (Peninsula Lab., Belniont, CA). [z]RIA (Phoenix, Europe, Kalsruhe, Germany). [æ]ELISA (Diagnostic Systems Laboratories, Webster, TX). [ø]Human Metabolic Hormone Magnetic Bead Panel (LINCOplex Kit, Millipore, St Louis, MO). [å]ELISA (Merck Millipore). Stars * denote that data was included in meta-analysis. # denotes that group characteristics was merged for the purpose of this analysis.
